# Incidence of football injuries sustained on artificial turf compared to grass and other playing surfaces: a systematic review and meta-analysis

**DOI:** 10.1016/j.eclinm.2023.101956

**Published:** 2023-04-13

**Authors:** Ilari Kuitunen, Ville Immonen, Oskari Pakarinen, Ville M. Mattila, Ville T. Ponkilainen

**Affiliations:** a Institute of Clinical Medicine and Department of Paediatrics, University of Eastern Finland, Kuopio, Finland; b Department of Paediatrics, Kuopio University Hospital, Kuopio, Finland; c Boys National Teams, The Finnish Football Association, Helsinki, Finland; d Faculty of Medicine and Health Technologies, Tampere University, Tampere, Finland; e Department of Orthopaedics and Traumatology, Tampere University Hospital, Tampere, Finland; f Department of Surgery, Central Finland Hospital Nova, Jyväskylä, Finland

**Keywords:** Football, Injury, Epidemiology, Incidence, Playing surface, Athletes, Sports medicine

## Abstract

**Background:**

Prior reviews have not conducted statistical synthesis of injury incidence on artificial turf in football. To analyse and compare the incidence of injuries sustained playing football (soccer) on artificial turf compared to grass and other playing surfaces.

**Methods:**

This was a systematic review and meta-analysis. We searched PubMed, Scopus, SPORTDiscus, and Web of Science databases in October 2022 without filters. All observational studies (prospective or retrospective) that analysed injuries sustained playing football on artificial turf and which included a control group that played on grass or other surface were included. Studies were included if they reported the number of injuries and the exposure time for the playing surfaces. Risk of bias was assessed by Newcastle-Ottawa Scale. A random effects model was used to calculate the pooled incidence rate ratios (IRR) with 95% confidence intervals. Protocol was registered with PROSPERO on October 30th, 2022. Registration number: CRD42022371414.

**Findings:**

We screened 1447 studies, and evaluated 67 full reports, and finally included 22 studies. Risk of bias was a notable issue, as only 5 of the 22 studies adjusted their analysis for potential confounders. Men (11 studies: IRR 0.82, CI 0.72–0.94) and women (5 studies: IRR 0.83, CI 0.76–0.91) had lower injury incidence on artificial turf. Professional players had a lower incidence of injury (8 studies: IRR 0.79, CI 0.70–0.90) on artificial turf, whereas there was no evidence of differences in the incidence of injury in amateur players (8 studies: IRR 0.91, CI 0.77–1.09). The incidence of pelvis/thigh (10 studies: IRR 0.72, CI 0.57–0.90), and knee injuries (14 studies: IRR 0.77, CI 0.64–0.92) were lower on artificial turf.

**Interpretation:**

The overall incidence of football injuries is lower on artificial turf than on grass. Based on these findings, the risk of injury can't be used as an argument against artificial turf when considering the optimal playing surface for football.

**Funding:**

No specific funding was received for this study.

**Research in context**

**Evidence before this study**

The safety of artificial turf as playing ground has been under debate since the first generation of artificial turf was introduced in 1960s. We searched PubMed and Scopus for words football and injuries and “artificial turf” without additional limitations to understand the prior literature in September and October 2022. Previous studies have reported contradictory results on varying from lower to similar to higher risk of injuries on artificial turf. However, we did not identify any previous systematic review which would have focused football injuries on artificial turf and provided statistical synthesis. Previous systematic reviews and meta-analyses had included all sports played on artificial turf and found higher injury incidence when American football was included, and that female athletes have higher incidence of anterior cruciate ligament injuries.

**Added value of this study**

This is the first systematic review that also produced statistical pooled synthesis on the football injury incidence on artificial turf compared to grass and other playing surfaces and by far the largest research reporting subgroups and all types of injuries. The overall incidence was 14% (7%–21%) lower on artificial turf than on grass. Men and women both had lower injury incidences on artificial turf. We did not find any evidence from any subgroup and injury category analysis that would have shown increased injury incidence on artificial turf. Furthermore, injuries to lower body (pelvis/thigh, and knees) had lower incidence on artificial turf.

**Implications of all the available evidence**

Based on these results, artificial turf seem to be safe surfaces for football as the overall injury incidence is low. Further studies especially in amateurs, women, and youth athletes are needed to have better estimates in these groups on the injury incidences. These findings can be utilized by sports physicians in everyday work but also by policy makers deciding on football pitch renovations and projects, and football associations when discussing optimal playing surfaces.

## Introduction

Football (soccer) is the most played team sport globally, and it is the national sport in many countries. Football has a major impact on communities both physically and financially.^1^ Traditionally, football has been played on natural surfaces such as grass. However, since the introduction of first-generation artificial turf in the 1960's, artificial surfaces have gained increasing popularity, especially recently. The quality of artificial turf has improved greatly in recent years. Currently, the International Association of Football Federations (FIFA) is implementing quality programs for artificial turf and artificial pitches may soon be awarded FIFA quality or quality pro standards.^2^ The main benefits of artificial turf are that it is easy to maintain and provides a flat surface, which is especially important in areas where the growing season is short due to the cold climate. An added benefit is that artificial turf does not require sunlight (easier to maintain in large stadia) and watering (saves water in dry areas).

However, since the introduction of first generation artificial turf, a key question has been whether the turf is associated with an increased or decreased incidence of injury.^3^ When injuries occur to top level players on artificial turf, they tend to make headlines. For example, AS Roma head coach Jose Mourinho claimed that playing on an artificial pitch in Norway caused a knee injury to a Roma player.^4^ A previous meta-analysis, which included all sports played on artificial turf, found that the rates of anterior cruciate ligament injuries were higher in women, but not in men.^5^ Interestingly, according to the findings of a novel meta-analysis,^6^ hamstring injuries are 50% more likely to occur on grass than on artificial turf in all field sports. Another recent systematic review reported that the risk of injury playing football on both playing surfaces was similar, but the authors did not conduct a statistical synthesis of the results.^7^ To date, the majority of the prior literature on injuries sustained on artificial turf has focused solely on American football. However, as it is known that football and American football are vastly different sports with different injury profiles, it is important that football is analysed separately.^8^^,^^9^

The aim of this systematic review and meta-analysis is to analyse the risk of injuries when playing football on artificial turf compared to grass and other playing surfaces.

## Methods

### Search strategy and selection criteria

We conducted a systematic review and meta-analysis. We searched the EBSCOhost (SPORTDsicus), PubMed, Scopus, and Web of Science databases in October 2022 using the following search phrase: Artificial AND (turf OR grass). Grey literature was not searched. Complete search strategy is provided in the Supplementary file S2. The search results were then uploaded to Covidence software (Alfred Health, Monash University, Melbourne, Australia) for screening. Two authors (IK and VI) independently screened the titles and abstracts and later the full texts. Cases of discrepancy were solved by reaching consensus. The screening process had moderate inter-rater reliability scores (proportionate agreement 0.96 and Cohen's Kappa 0.63).

To be included in the systematic review, a study had to fulfil all the following criteria. The study had to focus on football (soccer) only or report football separately. Further, injuries sustained on artificial turf had to be compared to injuries sustained on grass or other playing surfaces. We included prospective and retrospective observational (cohort) studies reporting the number of injuries per exposure time. If a study did only report the injury incidence without number of injuries or exposure time, it was excluded. Studies that did not report original data (editorials, reviews, systematic reviews, commentaries) were excluded. Studies not reported in English were also excluded. Conference presentations were excluded, but any corresponding published publications were hand searched, if not included in the initial search.

### Data analysis

Data extraction was performed by a single author (OP or VI) and verified by a second author (IK) to a pre-designed Excel spreadsheet to minimise potential extraction errors. We extracted the following information: name of authors, name of journal, publication year, country, study design, number of injuries, exposure time, injury types, level of play, sex, and comparator surface. Furthermore, exposure time was extracted either per hour or per athlete exposure. Athlete exposure was used only in one study, and it meant that a single player had attended either a training session or a game (Table 1). In studies that reported injuries per game (one study; Table 1), we estimated the incidence per playing hour by multiplying the number of games by eleven players per team and a playing time of 90 min to obtain the total number of exposure hours.

**Table 1:** Background information of the included studies.

Study	Country	Study period	Prospective or retrospective	Study design	Level of play	Turf generation	Age	Gender	Training or match	Total n of injuries	Exposure measure	Injury type
Almutawa 2014	Saudi-Arabia	2010–2011	Prospective	Cohort	Professional	Third	Adult	Men	Both	82	Hours	All injuries
Aoki 2010	Japan	2005	Prospective	Cohort	Amateur	Not specified	Youth	Both	Both	525	Hours	All injuries
Bjørneboe 2010	Norway	2004–2007	Prospective	Cohort	Professional	Third	Adult	Men	Both	1067	Hours	All injuries
Calloway 2019	USA	2013–2016	Retrospective	Cohort	Professional	Third	Adult	Men	Match	2147	Games	All injuries
Ekstrand 2006	Europe	2003–2004	Prospective	Cohort	Professional	Third	Adult	Men	Both	775	Hours	All injuries
Ekstrand 2011a	Europe	2001–2009	Prospective	Cohort	Professional	Third	Adult	Men	Both	2908	Hours	Muscle injuries
Ekstrand 2011b	Europe	2003–2008	Prospective	Cohort	Professional	Third	Adult	Both	Both	2105	Hours	Acute injuries
Ekstrand 2012	Europe	2001–2009	Prospective	Cohort	Professional	Third	Adult	Men	Both	51	Hours	Stress fractures
Fuller 2007a	USA	2005–2006	Prospective	Cohort	Amateur	Third	Adult	Both	Training	1592	Hours	All injuries
Fuller 2007b	USA	2005–2006	Prospective	Cohort	Amateur	Third	Adult	Both	Match	1794	Hours	All injuries
Howard 2020	USA	2004–2014	Retrospective	Cohort	Amateur	Not specified	Adult	Both	Both	3449	Athlete exposure	ACL
Hägglund 2011	Europe	2001–2009	Prospective	Cohort	Professional	Third	Adult	Men	Both	137	Hours	Patellar tendon injuries
Hägglund 2016	Sweden	2009	Prospective	Cohort	Amateur	Third	Youth	Women	Both	96	Hours	Knee injuries
Kordi 2011	Iran	2008	Prospective	Cohort	Amateur	Second	Adult	Men	Match	97	Hours	All injuries
Kristenson 2013	Norway, Sweden	2010–2011	Prospective	Cohort	Professional	Third	Adult	Men	Both	1020	Hours	Acute injuries
Kristenson 2016	Norway, Sweden	2010–2011	Prospective	Cohort	Professional	Third	Adult	Men	Both	372	Hours	All injuries
Lanzetti 2017	Italy	2011–2012	Prospective	Cohort	Professional	Third	Adult	Men	Match	43	Hours	All injuries
Meyers 2013	USA	2007–2011	Prospective	Cohort	Amateur	Third	Adult	Women	Match	693	Hours	All injuries
Meyers 2014	USA	2007–2012	Prospective	Cohort	Amateur	Third	Adult	Men	Match	722	Hours	All injuries
Rössler 2017	Switzerland, Czech Republic	2012–2014	Prospective	Cohort	Amateur	Not specified	Youth	Both	Both	417	Hours	All injuries
Soligard 2010	Norway	2005–2008	Prospective	Cohort	Amateur	Third	Youth	Both	Match	2454	Hours	Acute injuries
Steffen 2007	Norway	2005	Prospective	Cohort	Amateur	Third	Youth	Women	Both	456	Hours	Acute injuries

Risk of bias was assessed according to the Newcastle-Ottawa Scale for cohort studies.^10^ Two authors (IK and VI) independently conducted the assessments and conflicting cases were decided by mutual consensus.

All analyses were conducted according to the Cochrane handbook guidelines. To be pooled together in the meta-analysis, studies had to report the number of injuries per exposure time. If the exposure time and incidences were reported, the number of injuries were calculated. Similarly, if the number of injuries and incidence were reported, the exposure time was calculated.

Pooled injury incidence rate ratios (IRR) with 95% confidence intervals were calculated by mixed-effects Poisson regression model with random study effects. Heterogeneity was expected to be high due to the attributable factors of different players. Such factors included the physical testing results of the player, history of injury, and external factors such as weather, type of stud and playing surface interaction. Thus, for all analyses, a random effects method was used. To control the heterogeneity, we conducted more specific subgroup analyses with less expected heterogeneity. Statistical heterogeneity was assessed by the I^2^ statistic and is presented alongside the analyses in the forest plots. We performed sensitivity analysis by including only studies with the lowest risk of bias and another sensitivity analysis by including only prospective studies. Presence of publication bias was assessed by generating funnel plots and performing Egger's test. A further moderator analysis was performed by meta-regression to estimate the impact of publication year to IRR estimates.

Based on the previously published literature, we performed subgroup analyses because we expected the risk of injury to differ in certain scenarios. Thus, we compared the injury incidence rate ratio on artificial turf versus grass separately for men, women, training sessions, matches, amateur players, professional players, youth players (age less than 18), adult players, injury mechanisms, anatomical injury locations, and geographical location (Northern-Europe vs Central Europe, East-Asia, and the USA vs Middle-East). Additional sensitivity analysis was performed by including only studies analysing the latest (third) generation artificial turf.

We have rated the evidence quality for main outcomes according to the Grading of Recommendations, Assessment, Development and Evaluations (GRADE) framework.^11^ The evidence quality was ranked in a scale from very low to high.

This study has been reported according to the preferred reporting items in systematic reviews and meta-analysis (PRISMA) 2020. The PRISMA checklist is provided in Supplementary file S1.^12^

This systematic review was registered with PROSPERO (Registration number: CRD42022371414).

### Role of the funding source

There was no funding source for this study.

## Results

### Search results

**Fig. 1:**
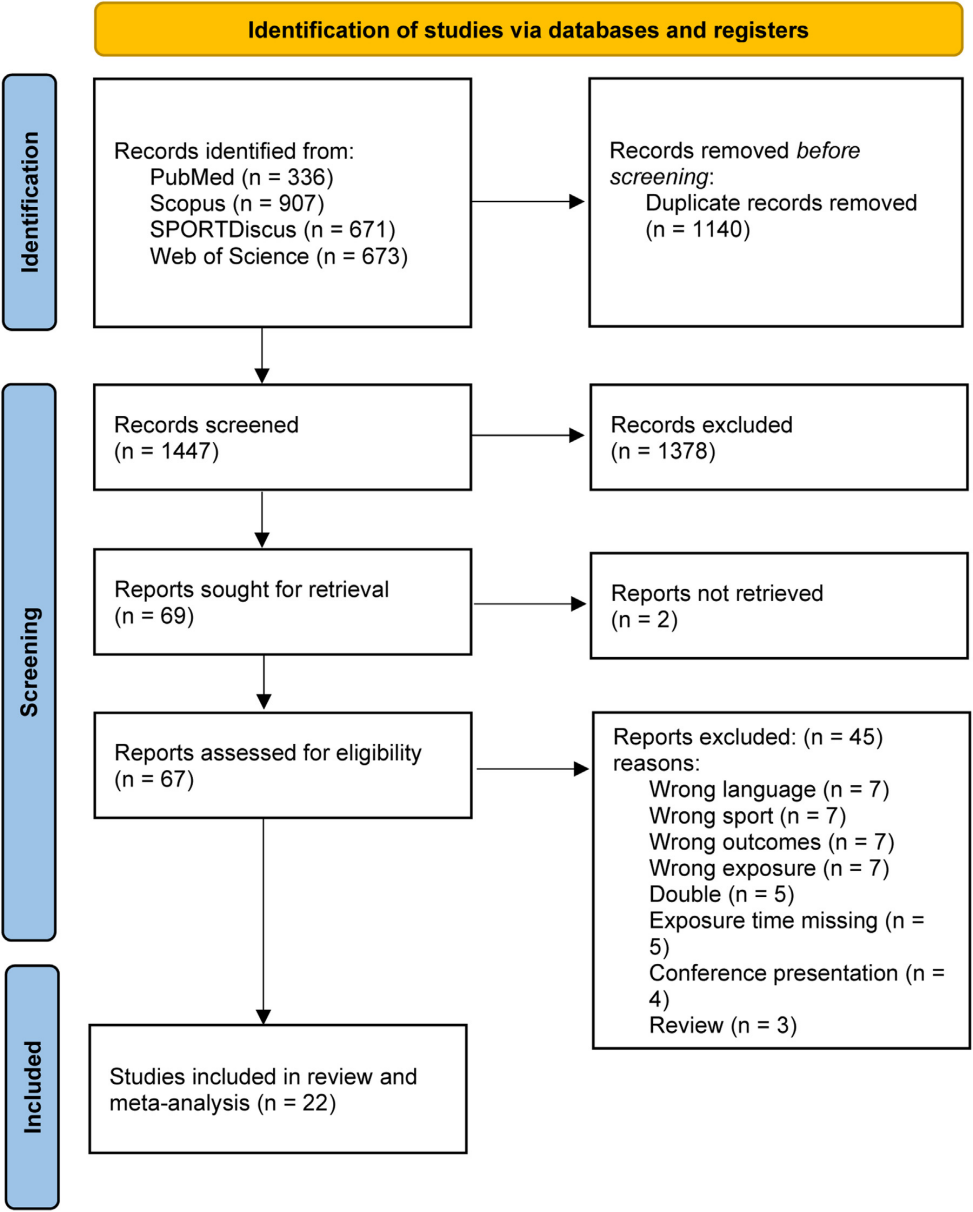
Flowchart of the study selection process.

Initially, a total of 1447 abstracts and titles were screened. In addition, we analysed 67 full reports and finally included 22 studies for systematic review and meta-analysis (Fig. 1).^13^, ^14^, ^15^, ^16^, ^17^, ^18^, ^19^, ^20^, ^21^, ^22^, ^23^, ^24^, ^25^, ^26^, ^27^, ^28^, ^29^, ^30^, ^31^, ^32^, ^33^ All the included studies were cohort studies. Of these, thirteen were conducted in Europe, six in the USA, and three in Asia (Table 1). All studies were conducted between 2001 and 2014. 16 studies focused on professional football players and 17 studies focused on adults. The number of injuries reported varied between 51 and 3449. One study was conducted on second generation turf, three studies did not specify the generation, and the rest 18 studies analysed third generation turf (Table 1).

### Risk of bias

The risk of bias in the included studies was mostly due to a failure to control for potential confounders in the analyses (Table 2). Indeed, only five studies tried to confound for potential sources of extrinsic bias and player attributable bias in their analyses. However, we judged that none of the included studies had to be removed from the analysis due to a high risk of bias.

**Table 2:** Risk of bias of the included studies assessed by Newcastle-Ottawa Scale. Maximum number of points is nine, and a higher score means the least risk of bias.

Study	Selection				Comparability	Outcome			Total
	Representativeness of the exposed cohort	Selection of the non- exposed cohort	Ascertainment of exposure	Demonstration that outcome of interest was not present at start of study	Comparability of cohorts based on the design or analysis	Assessment of outcome	Was follow-up long enough for outcomes to occur	Adequacy of follow up of cohorts	Total (9 max)
Almutawa 2014	1	1	1	1	0	1	1	1	7
Aoki 2010	1	1	1	1	0	1	1	1	7
Bjørneboe 2010	1	1	1	1	0	1	1	1	7
Calloway 2019	1	1	1	1	0	1	1	1	7
Ekstrand 2006	1	1	1	1	0	1	1	1	7
Ekstrand 2011a	1	1	1	1	0	1	1	1	7
Ekstrand 2011b	1	1	1	1	0	1	1	1	7
Ekstrand 2012	1	1	1	1	0	1	1	1	7
Fuller 2007a	1	1	1	1	0	1	1	1	7
Fuller 2007b	1	1	1	1	0	1	1	1	7
Howard 2020	1	1	0	1	0	1	1	1	6
Hägglund 2011	1	1	1	1	0	1	1	1	7
Hägglund 2016	1	1	1	1	2	1	1	1	9
Kordi 2011	1	1	1	1	0	1	1	1	7
Kristenson 2013	1	1	1	1	0	1	1	1	7
Kristenson 2016	1	1	1	1	0	1	1	1	7
Lanzetti 2017	1	1	1	1	0	1	1	1	7
Meyers 2013	1	1	1	1	2	1	1	1	9
Meyers 2014	1	1	1	1	2	1	1	1	9
Rössler 2018	1	1	1	1	2	1	1	1	9
Soligard 2012	1	1	1	1	2	1	1	1	9
Steffen 2007	1	1	1	1	0	1	1	1	7

### Overall injury incidences

**Fig. 2:**
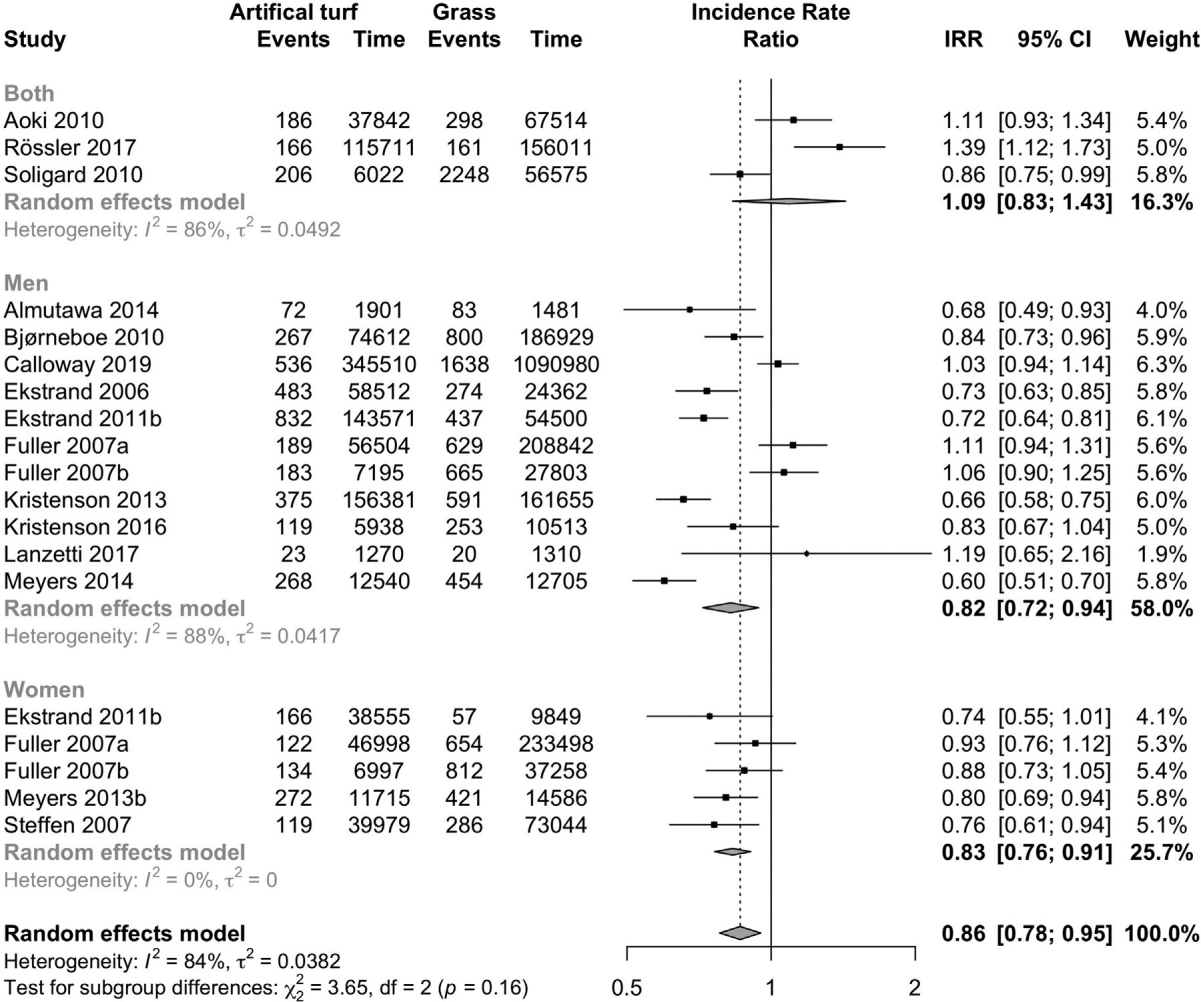
Forest plot of the incidence of overall injuries on artificial turf compared to grass stratified by sex.

**Fig. 3:**
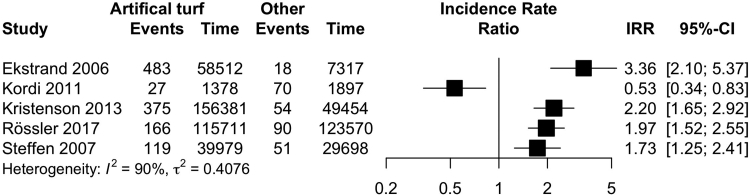
Forest plot of the incidence rate ratios of overall injuries on artificial turf compared to other playing surfaces.

**Fig. 4:**
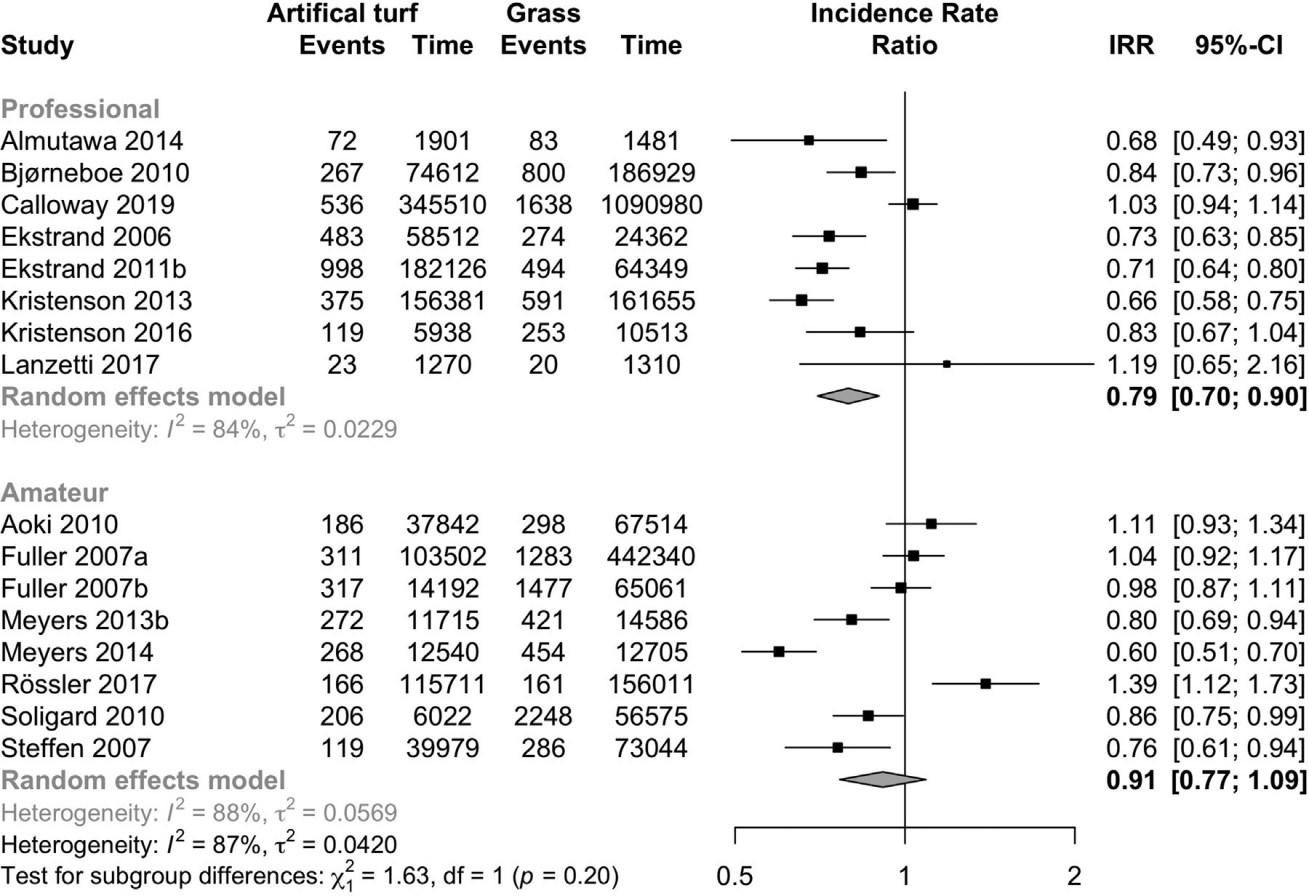
Forest plot of the injury incidence rate ratios on artificial turf compared to grass and other playing surfaces stratified between professional and amateur players.

**Fig. 5:**
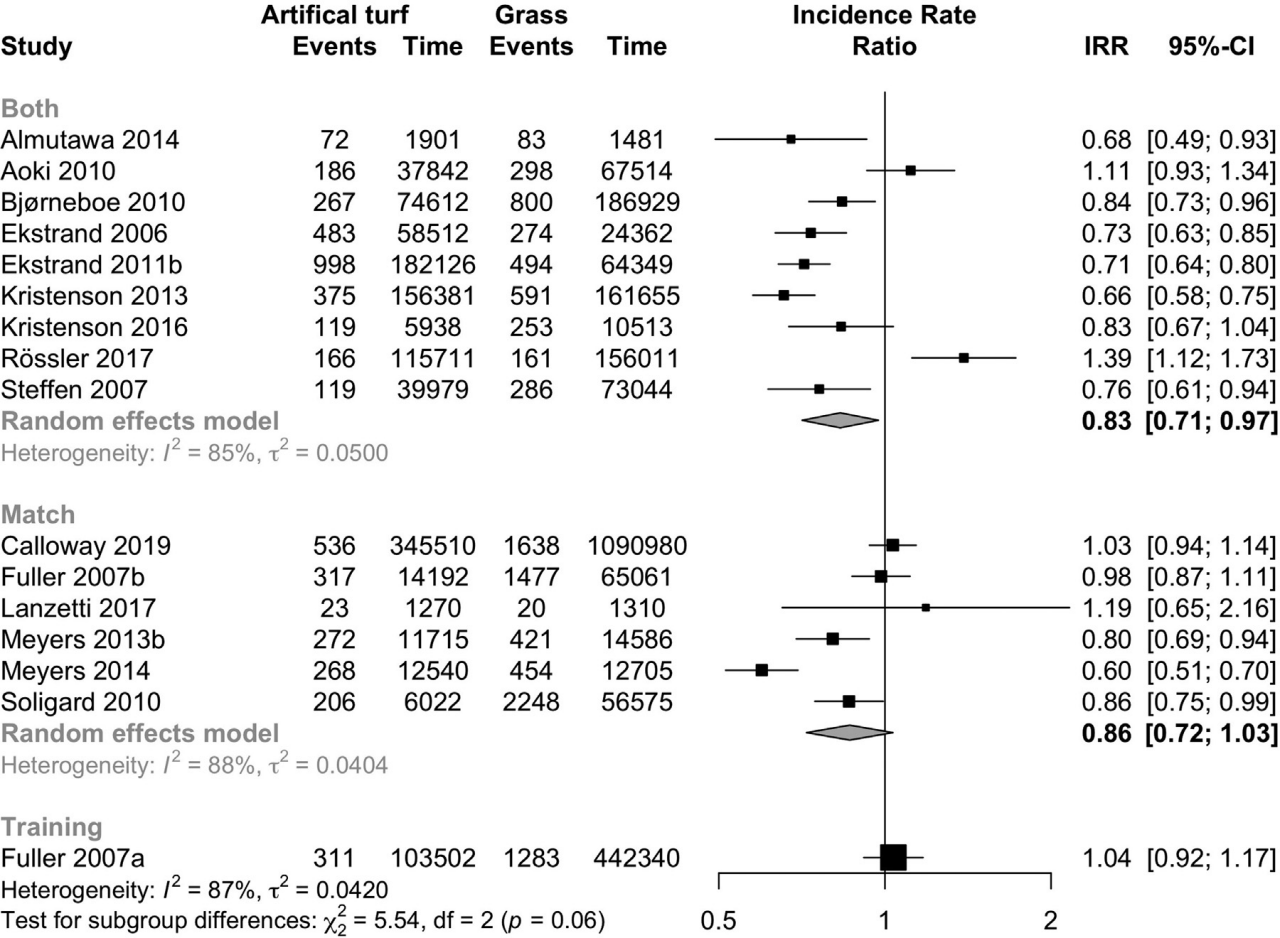
Forest plot of the injury incidence rate ratios on artificial turf compared to grass and other playing surfaces stratified by matches and training sessions.

Overall, the incidence of injury was lower on artificial turf than on grass (20 studies; IRR 0.86, CI 0.78–0.95; I^2^ 84%, Fig. 2; evidence quality low; Table 3). The injury incidence was higher on artificial turf when compared to other playing surfaces (5 studies; IRR 1.73, CI 1.25–2.41; I^2^ 90%; Fig. 3; evidence quality very low; Table 3). Both men (11 studies: IRR 0.82, CI 0.72–0.94; I^2^ 88%; Fig. 2) and women (5 studies: IRR 0.83, CI 0.76–0.91; I^2^ 0%; Fig. 2) had a lower incidence of injuries on artificial turf (evidence quality low, Table 3). Professional players had a lower incidence of injury (8 studies: IRR 0.79, CI 0.70–0.90; I^2^ 84%; I Fig. 4; evidence quality low; Table 3) on artificial turf, but there was no evidence of a difference in amateur players (8 studies: IRR 0.91, CI 0.77–1.09; I^2^ 88%; Fig. 4; evidence quality very low; Table 3). There was no evidence of any difference reported in studies that analysed matches played on artificial turf (6 studies: IRR 0.86, CI 0.72–1.03; I^2^ 85%; Fig. 5; evidence quality very low; Table 3) or training sessions (1 study: IRR 1.04, CI 0.92–1.17; Fig. 5; evidence quality very low; Table 3).

**Table 3:** Evidence quality for main outcomes assessed according to the GRADE framework.

Outcome	GRADE	Comment
Overall injury incidence		
Artificial turf vs grass	Low	Downgraded due to risk of bias, inconsistency, upgraded due to lack of imprecision
Artificial turf vs other surfaces	Very low	Downgraded due to risk of bias, inconsistency and limited study sample
Men	Low	Downgraded due to risk of bias, inconsistency, upgraded due to lack of imprecision
Women	Low	Downgraded due to risk of bias, limited sample size, upgraded due to lack of imprecision and inconsistency.
Professionals	Low	Downgraded due to risk of bias, inconsistency, upgraded due to lack of imprecision
Amateurs	Very low	Downgraded due to risk of bias, inconsistency and imprecision.
Matches	Very low	Downgraded due to risk of bias, inconsistency and imprecision.
Training	Very low	Downgraded due to risk of bias, inconsistency and imprecision.
Injury mechanism		
Non-contact	Low	Downgraded due to risk of bias, imprecision, upgraded due to low inconsistency.
Contact	Very low	Downgraded due to risk of bias, inconsistency and imprecision.
Injury type		
Muscle strain	Low	Downgraded due to risk of bias, inconsistency, upgraded due to lack of imprecision.
Contusions	Low	Downgraded due to risk of bias, imprecision, upgraded due to low inconsistency,
Sprains	Very low	Downgraded due to risk of bias, inconsistency and imprecision.

### Injury mechanisms, types, and locations

**Fig. 6:**
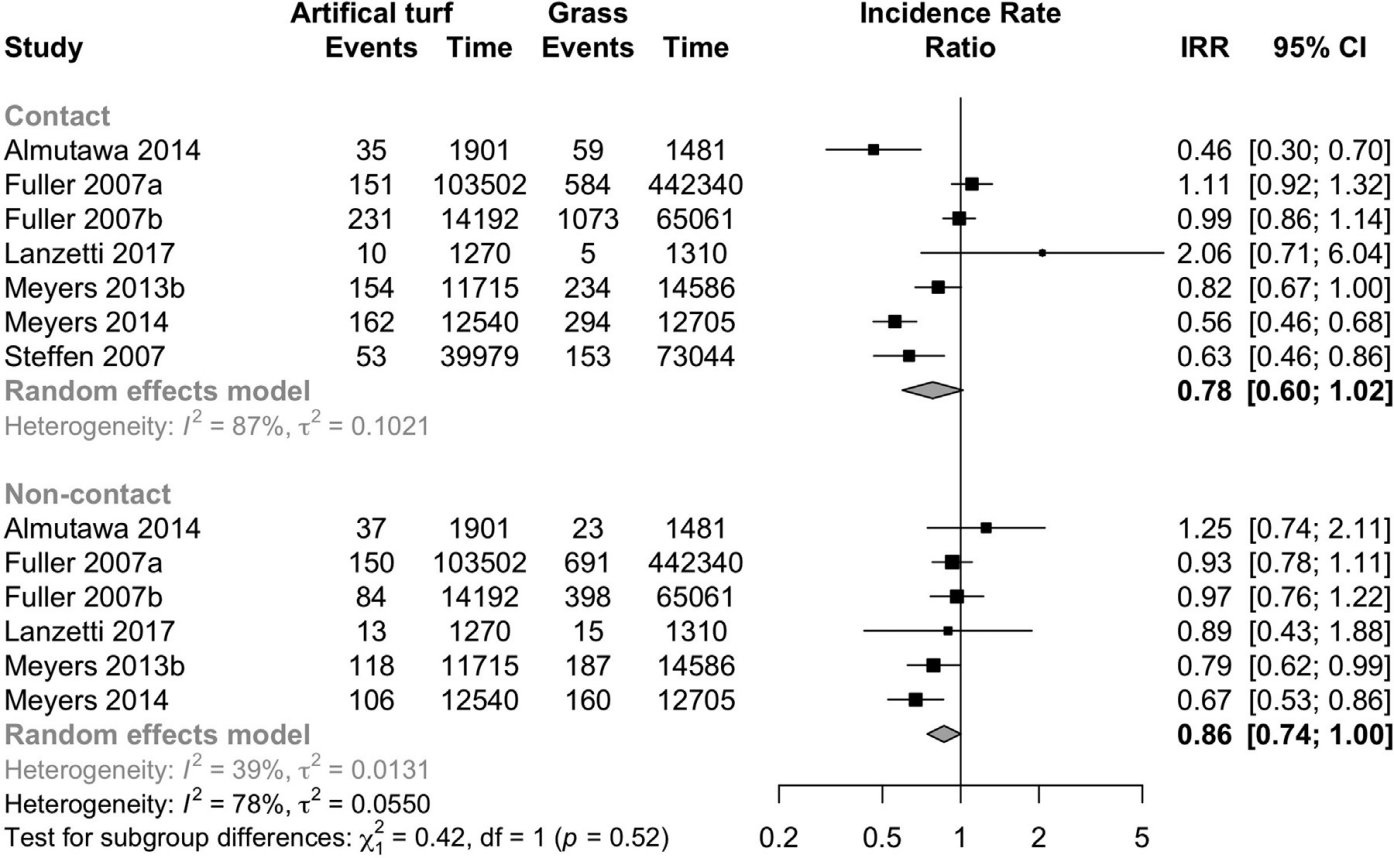
Forest plot of the injury incidence rate ratios on artificial turf compared to grass and other playing surfaces stratified by injury mechanism (contact vs non-contact).

**Fig. 7:**
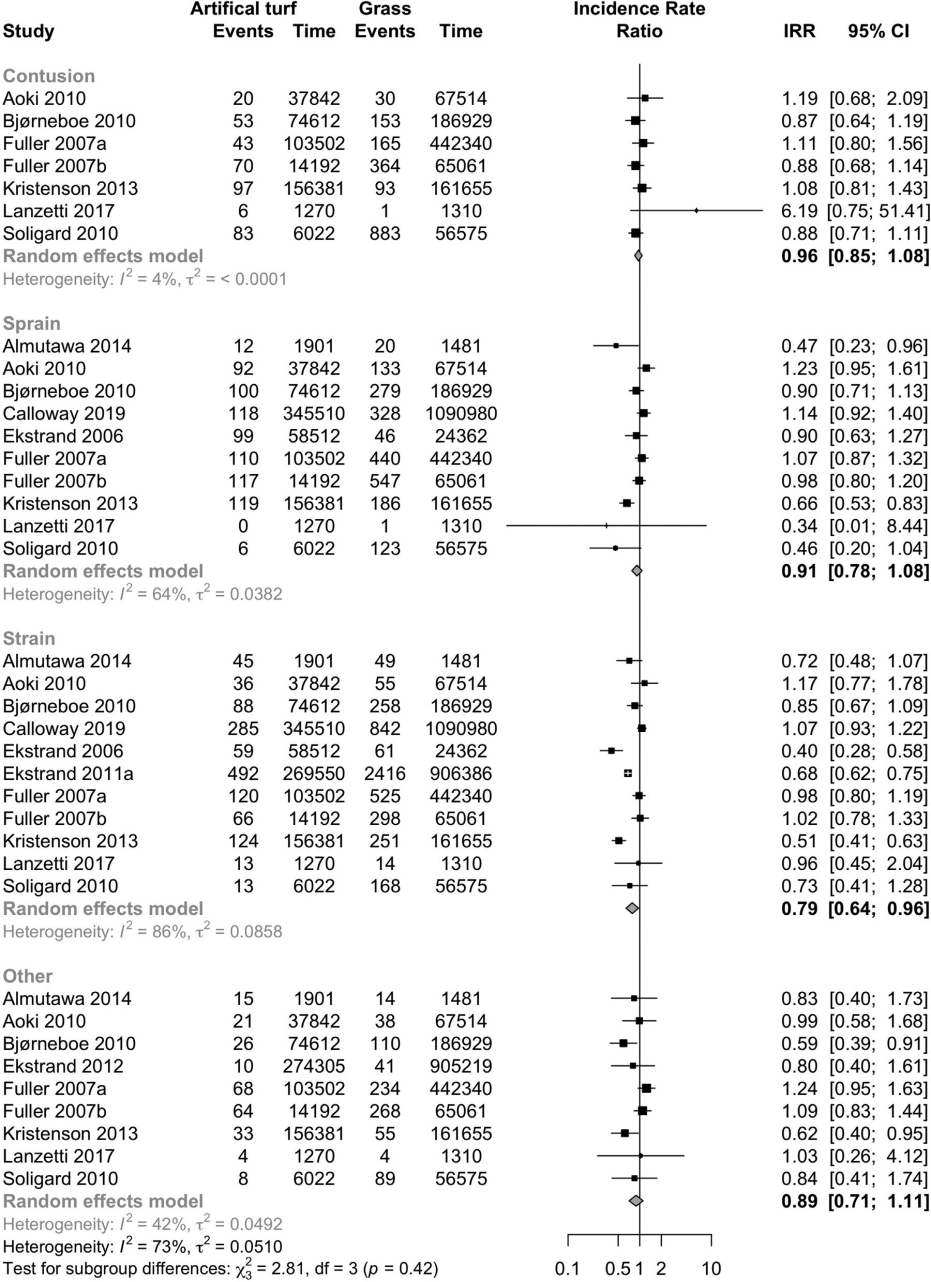
Forest plot of the injury incidence rate ratios on artificial turf compared to grass and other playing surfaces stratified by injury type (fracture, sprain, ligament injury).

Non-contact injuries were less frequent on artificial turf (6 studies: IRR 0.86, CI 0.74–1.00; I^2^ 39%; Fig. 6; evidence quality low, Table 3) than on grass. There was no evidence of differences in contact injuries (7 studies: IRR 0.78, CI 0.60–1.12; I^2^ 87%; Fig. 6; evidence quality very low, Table 3). Muscle strains were less frequent on artificial turf (11 studies: IRR 0.79, CI 0.64–0.96; I^2^ 86%; Fig. 7; Evidence quality low; Table 3), and other injury types (contusions, sprains, and other) did not show any evidence of differences between playing surfaces (Fig. 7, Table 3). In one study, stress fractures were assessed and the rates between the playing surfaces were similar (IRR 0.80, CI 0.40–1.61).

In a more specific analysis of the anatomical location of the injuries, the overall incidences of injury on artificial turf were lower for the total rate of lower body injuries (12 studies: IRR 0.86, CI 0.74–1.00; I^2^ 87%; Supplementary Figure S1), pelvis and thigh injuries (10 studies: IRR 0.72, CI 0.57–0.90; I^2^ 90%), and knee injuries (14 studies: IRR 0.77, CI 0.64–0.92; I^2^ 65%; Supplementary Figure S1). Furthermore, on artificial turf, men had a lower incidence of upper body (5 studies: IRR 0.73, CI 0.54–0.97; I^2^ 0%), pelvis and thigh (8 studies: IRR 0.70, CI 0.53–0.92; I^2^ 92%), and knee injuries (10 studies: IRR 0.76, CI 0.58–0.99; I^2^ 77%; Supplementary Figure S2). Furthermore, we found no evidence of differences in anatomical location in women (Supplementary Figure S3). Professional players had a lower incidence of head, upper body, lower body, knee, and pelvis injuries on artificial turf ((Supplementary Figure S4), whereas amateur players did not have an increased or decreased incidence of injury on artificial turf (Supplementary Figure S5). There were no differences in the incidences of injury between games or training sessions on artificial turf. However, a smaller number of studies analysed this difference (Supplementary Figures S6 and S7). Adult players had a lower incidence of lower body (10 studies: IRR 0.85, CI 0.73–0.99; I^2^ 87%), pelvis and thigh (8 studies: IRR 0.70, CI 0.53–0.92; I^2^ 92%), and knee injuries (11 studies: IRR 0.76, CI 0.61–0.94; I^2^ 73%; Supplementary Figure S8), but there were no evidence of any differences in incidences of injury in youth players (Supplementary Figure S9).

### Geographical location

In geographical analysis, one study was conducted in Middle East, and it found lower injury incidence on artificial turf (IRR 0.68, CI 0.49–0.93; Supplementary Figure S10). Ten studies were performed in Central regions (includes Central Europe, East-Asia, and the USA), and in these regions the estimates did not show evidence of a difference (IRR 0.91, CI 0.78–1.07; Supplementary Figure S10). Five studies were conducted in Northern Europe, and the injury incidence was lower on artificial turf (IRR 0.78, CI 0.70–0.87; Supplementary Figure S10).

### Sensitivity analyses and other additional analyses

In a sensitivity analysis with only third generation artificial turfs included, the incidence estimates did not show evidence of a difference compared to the main analyses in most of the analyses (Supplementary Figures S11, S13–S15). However, the estimate did change notably in amateurs, and the incidence was lower on artificial turf (IRR 0.83, CI 0.71–0.98; Supplementary Figure S12). Similarly, the additional sensitivity analysis with only prospective studies did not change notably any of the IRR estimates (Supplementary Figures S16–S20). A further sensitivity analysis, for which only studies with highest quality were included, did not change the effect estimates (Supplementary Figure S21). We performed a further meta-regression moderator analysis to estimate the impact of publication year and it did not find any meaningful associations in any of the main analyses. Publication bias was assessed by funnel plots and Egger's test and we did not find evidence of it (Supplementary Figure S22).

## Discussion

Based on the evidence from this systematic review, the incidence of injury is typically lower when football is played on artificial turf than it is when played on grass. This finding was seen in both men and women. Professional players had a lower incidence of injury on artificial turf, whereas amateur players had a similar incidence of injury on grass and other playing surfaces and artificial playing surfaces. Similarly, adult players had a lower incidence of injuries on artificial turf, but youth players did not. Non-contact injuries and muscle strains were less frequent on artificial turf. Furthermore, in subgroup analysis, the incidence of pelvis and thigh, and knee injuries sustained on artificial turf were found to be lower in men and professional players. The majority of the subgroups analyses had high uncertainty and imprecision in the estimates with wide confidence intervals.

To the best of our knowledge, this is the largest study on the incidence of injury associated with playing football on artificial turf. A recent meta-analysis by Xiao et al. found that women had a higher incidence of ACL injury in all sports played on artificial turf, but the incidences of injury were similar in men and in training sessions.^5^ In our analysis, we did not find any evidence of an increased incidence of knee or ACL injuries in women or in games. An earlier systematic review by Balazs et al. found an increased risk for ACL injury in American football, but not in football.^34^ From the results of our analysis, it seems that the overall incidence of knee injuries was lower on artificial turf. A systematic review by Gould et al., which did not present any quantitative pooled synthesis, concluded that a higher rate of foot and ankle injuries occur on artificial turf. However, the lack of a meta-analysis lessens the value of such a conclusion.^7^ In our analysis, no evidence that any joint had an increased risk for injuries on artificial turf was found. Overall, lower rates of non-contact injuries and strains occurred on artificial turf. A previous meta-analysis by Maniar et al. reported an increased hamstring injury risk in field sports played on grass compared to artificial turf.^6^ Similar findings were also seen in our results, as the incidence of pelvic and thigh region injuries were 27% lower on artificial turf than on grass.

Based on the finding of this study, the incidence of injury is lower on artificial turf, which should be noted when discussing and planning the renovation of football fields. Although football is traditionally played on grass, it seems that the flat and homogenous surface offered by artificial turf may prevent injuries, and thus reduce the use of resources and related healthcare costs. We performed a geographical stratified analysis to estimate indirectly the weather conditions, and it seemed that especially the incidence was lower in Northern Europe, where the growing season for grass is the shortest. Additionally, we analysed only the third generation artificial turfs and the injury incidences were mostly lower or similar to grass. When discussing the optimal playing surface and possible playing surfaces in football, possible injuries should not be used as an argument to prevent artificial turf being used. This was the case for the men's 2026 World Cup in the USA, where FIFA decided that all artificial turf pitches should be converted to grass prior to the World Cup. Interestingly, women played on artificial turf in the 2015 World Cup in Canada and youth World Cups have also been played on artificial turf. Furthermore, the official rules of both FIFA and UEFA allow artificial turf to be used as a playing surface.

A survey conducted with professional football coaches in the Netherlands revealed interesting results, as 63% of the participants saw artificial turf as the surface of the future, and 57% believed that technical skills improve better on artificial turf. However, 70% of participants still preferred natural grass.^35^ Professional players have reported a higher fear of injury when playing on artificial turf compared to grass.^36^

Recent studies, however, have shown that the players' preference for natural grass is more likely about cognitive bias rather than physical differences between the playing surfaces.^37^ Although elite level players were found to make less slide tackles and prefer shorter passes on artificial turf, the measured game parameters were otherwise similar.^38^ However, the players’ feelings were clearly more negative towards artificial turf.^38^

To the best of our knowledge, this is the largest study on the incidence of injury associated with playing football on artificial turf. Moreover, we are unaware of previous studies that provide pooled estimates of the differences in incidence of injury between different playing surfaces. The present study was conducted according to our study protocol, and we only made minor deviations from the original protocol. For example, we decided to use the Newcastle-Ottawa scale alone in reporting the risk of bias instead of the Joanna Briggs Institute Critical Appraisal checklist.

The limitations of the present study arise mainly from the included original studies. In many cases, injuries were defined differently between the studies. For example, some studies classified injury as any event that led to the interruption of a training session or match, whereas other studies defined injury as an event that required assessment from medical personnel (physiotherapist or team doctor). In another classification, an injury was defined as leading to absence from training sessions or matches. Although this causes heterogeneity between the studies, we pooled the incidence rate ratios, which means that the pooled estimate is derived from the interstudy comparisons. A further limitation was the failure to adjust for confounding, as 17 of the 22 studies did not control for external confounding factors, such as the weather, wetness of the pitch and the studs used, or control for player attributable confounders (history of injury, physical abilities, etc). A further limitation is the limited number of included studies in the subgroup analyses, which causes clear imprecision to the estimates.

Future research is still needed to better understand the epidemiology of injuries, especially in amateur and youth athletes playing on artificial turf. More research is also needed at the elite female level to better estimate the incidence of injury on artificial turf. Future studies should be designed to better control for potential player attributable and external confounding factors in the analyses to increase the quality in the reporting.

The results of our current study can be utilized in decision making when planning new football pitches both in professional level and in communities as the artificial turf seems to have lower injury incidence than grass pitches. Furthermore, these results can be utilized by medical departments in football teams and associations when discussing factors related to possible injuries.

## Contributors

Ilari Kuitunen: Conseptualization, Data curation, Investigation, Methodology, Validation, Writing—original draft, Writing—review & editing.

Ville Immonen: Conseptualization, Data curation, Validation, Writing—original draft, Writing—review & editing.

Oskari Pakarinen: Data curation, Validation, Writing—review & editing.

Ville M Mattila: Conseptualization, Funding acquisition, Project administration, Resources, Supervision, Writing—review & editing.

Ville T Ponkilainen: Formal analysis, Investigation, Methodology, Resources, Software, Validation, Visualization, Writing—review & editing.

## Data sharing statement

All data used in the analyses are available upon request from the corresponding author.

## Declaration of interests

None of the authors have any potential conflicts of interest.
