# Supplementary material for: Incidence of football injuries sustained on artificial turf compared to grass and other playing surfaces: a systematic review and meta-analysis
Source: eClinicalMedicine. 2023 Apr 13;59:101956. doi: 10.1016/j.eclinm.2023.101956 (PMC10139885; doi:10.1016/j.eclinm.2023.101956)
Supplement: Supplementary file S2 [file mmc2.docx]

**Supplementary file 2**

**Complete search strategy**

PubMed: ("artificial"[All Fields] OR "artificially"[All Fields]) AND ("turf"[All Fields] OR ("grassed"[All Fields] OR "poaceae"[MeSH Terms] OR "poaceae"[All Fields] OR "grass"[All Fields] OR "grasses"[All Fields]))

Scopus: TITLE-ABS-KEY ( artificial AND ( turf OR grass ) )

SPORTDiscus: Boolean/Phrase: artificial and (turf or grass) (ALL FIELDS)

Web of Science: artificial AND ( turf OR grass ) (ALL FIELDS)
