# Supplementary material for: Incidence of football injuries sustained on artificial turf compared to grass and other playing surfaces: a systematic review and meta-analysis
Source: eClinicalMedicine. 2023 Apr 13;59:101956. doi: 10.1016/j.eclinm.2023.101956 (PMC10139885; doi:10.1016/j.eclinm.2023.101956)

**Supplementary figures**

Supplementary figure 1: Forest plot of the injury incidence rate ratios on artificial turf compared to grass and other playing surfaces stratified by the anatomical location of the injury in both men and women.

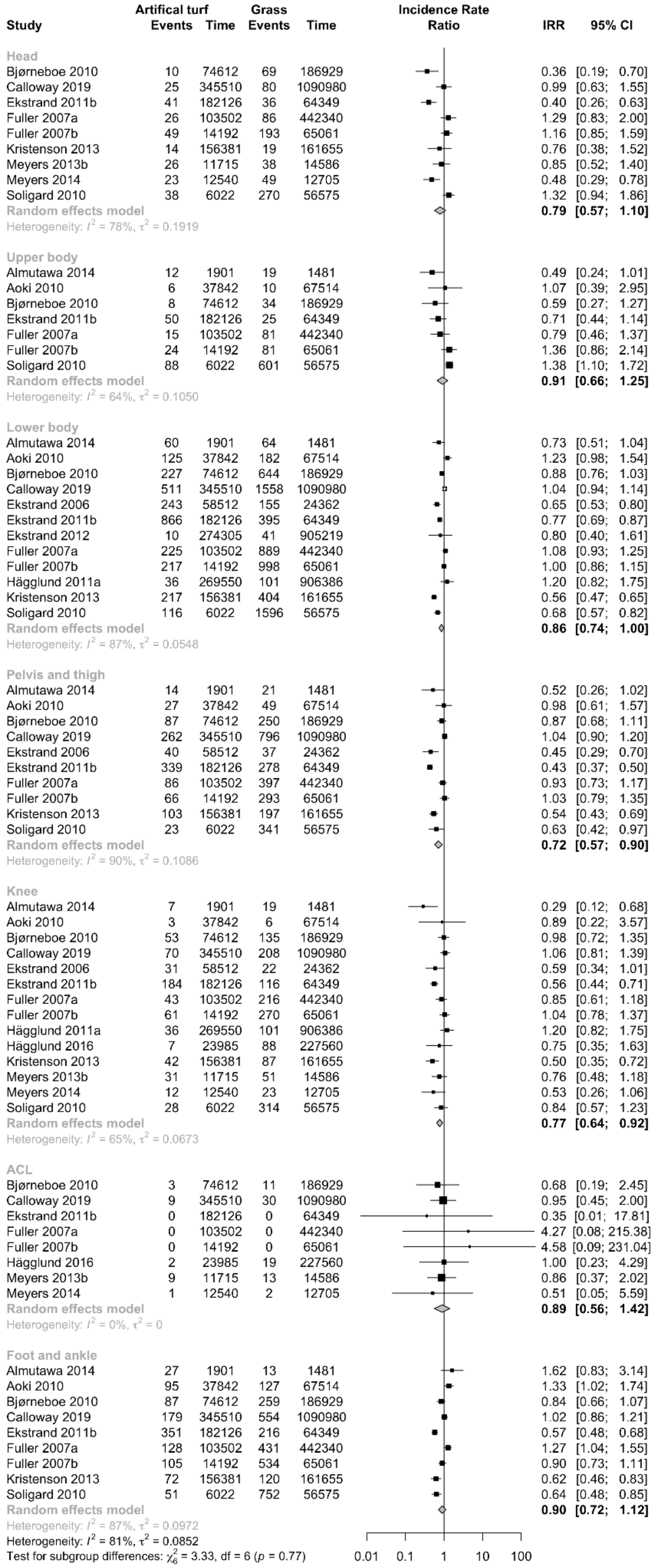

Supplementary figure 2: Forest plot of the injury incidence rate ratios on artificial turf compared to grass and other playing surfaces stratified by the anatomical location of the injury in men.

| Study | Artifical turf Events | Artifical turf Time | Grass Events | Grass Time | IRR | 95% CI |
|---|---|---|---|---|---|---|
| **Head** | | | | | | |
| Bjørneboe 2010 | 10 | 74612 | 69 | 186929 | 0.36 | [0.19; 0.70] |
| Calloway 2019 | 25 | 345510 | 80 | 1090980 | 0.99 | [0.63; 1.55] |
| Ekstrand 2011b | 30 | 143571 | 30 | 54500 | 0.38 | [0.23; 0.63] |
| Fuller 2007a | 11 | 56504 | 44 | 208842 | 0.92 | [0.48; 1.79] |
| Fuller 2007b | 31 | 7195 | 66 | 27803 | 1.82 | [1.18; 2.78] |
| Kristenson 2013 | 14 | 156381 | 19 | 161655 | 0.76 | [0.38; 1.52] |
| Meyers 2014 | 23 | 12540 | 49 | 12705 | 0.48 | [0.29; 0.78] |
| Random effects model | | | | | **0.71** | **[0.45; 1.12]** |
| Heterogeneity: $I^2$ = 82%, $\tau^2$ = 0.2914 | | | | | | |
| **Upper body** | | | | | | |
| Almutawa 2014 | 12 | 1901 | 19 | 1481 | 0.49 | [0.24; 1.01] |
| Bjørneboe 2010 | 8 | 74612 | 34 | 186929 | 0.59 | [0.27; 1.27] |
| Ekstrand 2011b | 44 | 143571 | 22 | 54500 | 0.76 | [0.46; 1.27] |
| Fuller 2007a | 9 | 56504 | 48 | 208842 | 0.69 | [0.34; 1.41] |
| Fuller 2007b | 12 | 7195 | 42 | 27803 | 1.10 | [0.58; 2.10] |
| Random effects model | | | | | **0.73** | **[0.54; 0.97]** |
| Heterogeneity: $I^2$ = 0%, $\tau^2$ = 0 | | | | | | |
| **Lower body** | | | | | | |
| Almutawa 2014 | 60 | 1901 | 64 | 1481 | 0.73 | [0.51; 1.04] |
| Bjørneboe 2010 | 227 | 74612 | 644 | 186929 | 0.88 | [0.76; 1.03] |
| Calloway 2019 | 511 | 345510 | 1558 | 1090980 | 1.04 | [0.94; 1.14] |
| Ekstrand 2006 | 243 | 58512 | 155 | 24362 | 0.65 | [0.53; 0.80] |
| Ekstrand 2011b | 723 | 143571 | 349 | 54500 | 0.79 | [0.69; 0.89] |
| Ekstrand 2012 | 10 | 274305 | 41 | 905219 | 0.80 | [0.40; 1.61] |
| Fuller 2007a | 138 | 56504 | 420 | 208842 | 1.21 | [1.00; 1.47] |
| Fuller 2007b | 123 | 7195 | 448 | 27803 | 1.06 | [0.87; 1.30] |
| Hägglund 2011a | 36 | 269550 | 101 | 906386 | 1.20 | [0.82; 1.75] |
| Kristenson 2013 | 217 | 156381 | 404 | 161655 | 0.56 | [0.47; 0.65] |
| Random effects model | | | | | **0.86** | **[0.73; 1.03]** |
| Heterogeneity: $I^2$ = 87%, $\tau^2$ = 0.0600 | | | | | | |
| **Pelvis and thigh** | | | | | | |
| Almutawa 2014 | 14 | 1901 | 21 | 1481 | 0.52 | [0.26; 1.02] |
| Bjørneboe 2010 | 87 | 74612 | 250 | 186929 | 0.87 | [0.68; 1.11] |
| Calloway 2019 | 262 | 345510 | 796 | 1090980 | 1.04 | [0.90; 1.20] |
| Ekstrand 2006 | 40 | 58512 | 37 | 24362 | 0.45 | [0.29; 0.70] |
| Ekstrand 2011b | 288 | 143571 | 258 | 54500 | 0.42 | [0.36; 0.50] |
| Fuller 2007a | 56 | 56504 | 196 | 208842 | 1.06 | [0.78; 1.42] |
| Fuller 2007b | 42 | 7195 | 174 | 27803 | 0.93 | [0.67; 1.31] |
| Kristenson 2013 | 103 | 156381 | 197 | 161655 | 0.54 | [0.43; 0.69] |
| Random effects model | | | | | **0.70** | **[0.53; 0.92]** |
| Heterogeneity: $I^2$ = 92%, $\tau^2$ = 0.1330 | | | | | | |
| **Knee** | | | | | | |
| Almutawa 2014 | 7 | 1901 | 19 | 1481 | 0.29 | [0.12; 0.68] |
| Bjørneboe 2010 | 53 | 74612 | 135 | 186929 | 0.98 | [0.72; 1.35] |
| Calloway 2019 | 70 | 345510 | 208 | 1090980 | 1.06 | [0.81; 1.39] |
| Ekstrand 2006 | 31 | 58512 | 22 | 24362 | 0.59 | [0.34; 1.01] |
| Ekstrand 2011b | 149 | 143571 | 107 | 54500 | 0.53 | [0.41; 0.68] |
| Fuller 2007a | 24 | 56504 | 90 | 208842 | 0.99 | [0.63; 1.55] |
| Fuller 2007b | 27 | 7195 | 86 | 27803 | 1.21 | [0.79; 1.87] |
| Hägglund 2011a | 36 | 269550 | 101 | 906386 | 1.20 | [0.82; 1.75] |
| Kristenson 2013 | 42 | 156381 | 87 | 161655 | 0.50 | [0.35; 0.72] |
| Meyers 2014 | 12 | 12540 | 23 | 12705 | 0.53 | [0.26; 1.06] |
| Random effects model | | | | | **0.76** | **[0.58; 0.99]** |
| Heterogeneity: $I^2$ = 77%, $\tau^2$ = 0.1305 | | | | | | |
| **ACL** | | | | | | |
| Bjørneboe 2010 | 3 | 74612 | 11 | 186929 | 0.68 | [0.19; 2.45] |
| Calloway 2019 | 9 | 345510 | 30 | 1090980 | 0.95 | [0.45; 2.00] |
| Meyers 2014 | 1 | 12540 | 2 | 12705 | 0.51 | [0.05; 5.59] |
| Random effects model | | | | | **0.84** | **[0.45; 1.57]** |
| Heterogeneity: $I^2$ = 0%, $\tau^2$ = 0 | | | | | | |
| **Foot and ankle** | | | | | | |
| Almutawa 2014 | 27 | 1901 | 13 | 1481 | 1.62 | [0.83; 3.14] |
| Bjørneboe 2010 | 87 | 74612 | 259 | 186929 | 0.84 | [0.66; 1.07] |
| Calloway 2019 | 179 | 345510 | 554 | 1090980 | 1.02 | [0.86; 1.21] |
| Ekstrand 2011b | 294 | 143571 | 199 | 54500 | 0.56 | [0.47; 0.67] |
| Fuller 2007a | 81 | 56504 | 219 | 208842 | 1.37 | [1.06; 1.76] |
| Fuller 2007b | 62 | 7195 | 253 | 27803 | 0.95 | [0.72; 1.25] |
| Kristenson 2013 | 72 | 156381 | 120 | 161655 | 0.62 | [0.46; 0.83] |
| Random effects model | | | | | **0.90** | **[0.69; 1.17]** |
| Heterogeneity: $I^2$ = 87%, $\tau^2$ = 0.1050 | | | | | | |

Heterogeneity: $I^2$ = 83%, $\tau^2$ = 0.1037

Test for subgroup differences: $\chi^2_6$ = 3.34, df = 6 ($p$ = 0.77)

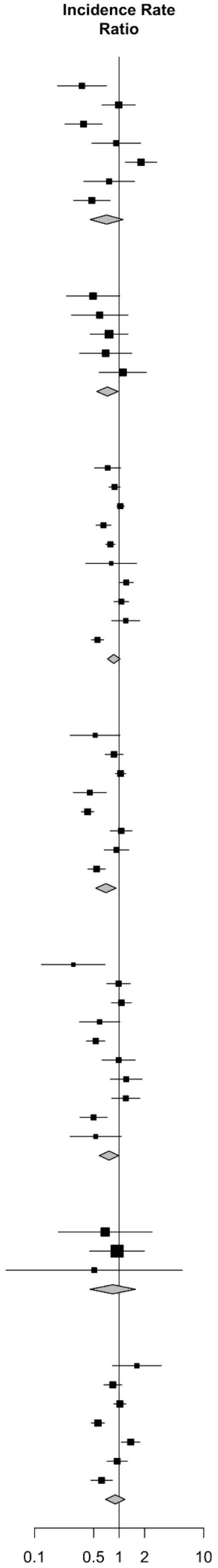

Supplementary figure 3: Forest plot of the injury incidence rate ratios on artificial turf compared to grass and other playing surfaces stratified by the anatomical location of the injury in women.

| Study | Artifical turf Events | Artifical turf Time | Grass Events | Grass Time | IRR | 95% CI |
|---|---|---|---|---|---|---|
| **Head** | | | | | | |
| Ekstrand 2011b | 11 | 38555 | 6 | 9849 | 0.47 | [0.17; 1.27] |
| Fuller 2007a | 15 | 46998 | 42 | 233498 | 1.77 | [0.98; 3.20] |
| Fuller 2007b | 18 | 6997 | 127 | 37258 | 0.75 | [0.46; 1.24] |
| Meyers 2013b | 26 | 11715 | 38 | 14586 | 0.85 | [0.52; 1.40] |
| **Random effects model** | | | | | **0.91** | **[0.56; 1.47]** |
| Heterogeneity: $I^2 = 59\%, \tau^2 = 0.1373$ | | | | | | |
| **Upper body** | | | | | | |
| Ekstrand 2011b | 6 | 38555 | 3 | 9849 | 0.51 | [0.13; 2.04] |
| Fuller 2007a | 6 | 46998 | 33 | 233498 | 0.90 | [0.38; 2.16] |
| Fuller 2007b | 12 | 6997 | 39 | 37258 | 1.64 | [0.86; 3.13] |
| **Random effects model** | | | | | **1.10** | **[0.60; 2.03]** |
| Heterogeneity: $I^2 = 27\%, \tau^2 = 0.0853$ | | | | | | |
| **Lower body** | | | | | | |
| Ekstrand 2011b | 143 | 38555 | 46 | 9849 | 0.79 | [0.57; 1.11] |
| Fuller 2007a | 87 | 46998 | 469 | 233498 | 0.92 | [0.73; 1.16] |
| Fuller 2007b | 94 | 6997 | 550 | 37258 | 0.91 | [0.73; 1.13] |
| **Random effects model** | | | | | **0.89** | **[0.77; 1.03]** |
| Heterogeneity: $I^2 = 0\%, \tau^2 = 0$ | | | | | | |
| **Pelvis and thigh** | | | | | | |
| Ekstrand 2011b | 51 | 38555 | 20 | 9849 | 0.65 | [0.39; 1.09] |
| Fuller 2007a | 30 | 46998 | 201 | 233498 | 0.74 | [0.51; 1.09] |
| Fuller 2007b | 24 | 6997 | 119 | 37258 | 1.07 | [0.69; 1.67] |
| **Random effects model** | | | | | **0.81** | **[0.61; 1.07]** |
| Heterogeneity: $I^2 = 19\%, \tau^2 = 0.0105$ | | | | | | |
| **Knee** | | | | | | |
| Ekstrand 2011b | 35 | 38555 | 9 | 9849 | 0.99 | [0.48; 2.07] |
| Fuller 2007a | 19 | 46998 | 126 | 233498 | 0.75 | [0.46; 1.21] |
| Fuller 2007b | 34 | 6997 | 184 | 37258 | 0.98 | [0.68; 1.42] |
| Hägglund 2016 | 7 | 23985 | 88 | 227560 | 0.75 | [0.35; 1.63] |
| Meyers 2013b | 31 | 11715 | 51 | 14586 | 0.76 | [0.48; 1.18] |
| **Random effects model** | | | | | **0.85** | **[0.68; 1.06]** |
| Heterogeneity: $I^2 = 0\%, \tau^2 = 0$ | | | | | | |
| **ACL** | | | | | | |
| Hägglund 2016 | 2 | 23985 | 19 | 227560 | 1.00 | [0.23; 4.29] |
| Meyers 2013b | 9 | 11715 | 13 | 14586 | 0.86 | [0.37; 2.02] |
| **Random effects model** | | | | | **0.89** | **[0.43; 1.86]** |
| Heterogeneity: $I^2 = 0\%, \tau^2 = 0$ | | | | | | |
| **Foot and ankle** | | | | | | |
| Ekstrand 2011b | 57 | 38555 | 17 | 9849 | 0.86 | [0.50; 1.47] |
| Fuller 2007a | 47 | 46998 | 212 | 233498 | 1.10 | [0.80; 1.51] |
| Fuller 2007b | 43 | 6997 | 281 | 37258 | 0.81 | [0.59; 1.12] |
| **Random effects model** | | | | | **0.93** | **[0.74; 1.17]** |
| Heterogeneity: $I^2 = 0\%, \tau^2 = 0.0053$ | | | | | | |

Heterogeneity: $I^2 = 0\%, \tau^2 = 0$

Test for subgroup differences: $\chi^2_6 = 1.22$, df = 6 ($p = 0.98$)

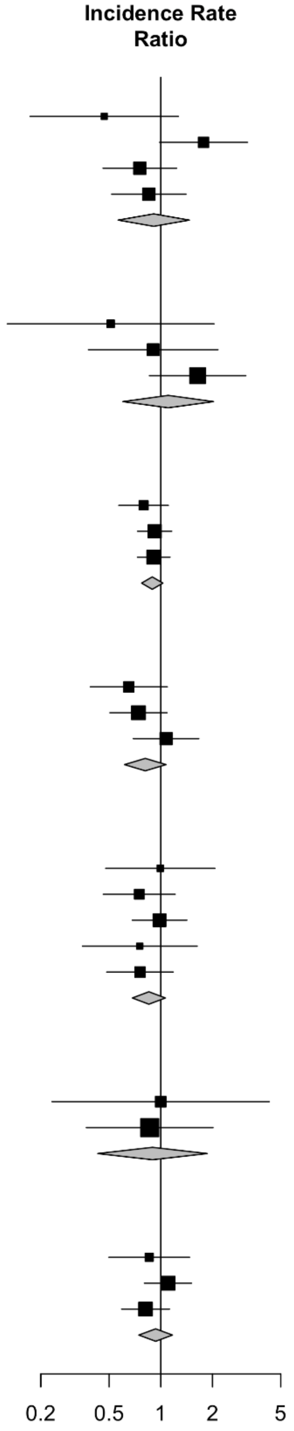

Supplementary figure 4: Forest plot of the injury incidence rate ratios on artificial turf compared to grass and other playing surfaces stratified by the anatomical location of the injury in professional players.

| Study | Artifical turf Events | Artifical turf Time | Grass Events | Grass Time | IRR | 95% CI |
|---|---|---|---|---|---|---|
| **Head** | | | | | | |
| Bjørneboe 2010 | 10 | 74612 | 69 | 186929 | 0.36 | [0.19; 0.70] |
| Calloway 2019 | 25 | 345510 | 80 | 1090980 | 0.99 | [0.63; 1.55] |
| Ekstrand 2011b | 41 | 182126 | 36 | 64349 | 0.40 | [0.26; 0.63] |
| Kristenson 2013 | 14 | 156381 | 19 | 161655 | 0.76 | [0.38; 1.52] |
| Random effects model | | | | | **0.58** | **[0.35; 0.95]** |
| Heterogeneity: $I^2 = 71\%$, $\tau^2 = 0.1722$ | | | | | | |
| **Upper body** | | | | | | |
| Almutawa 2014 | 12 | 1901 | 19 | 1481 | 0.49 | [0.24; 1.01] |
| Bjørneboe 2010 | 8 | 74612 | 34 | 186929 | 0.59 | [0.27; 1.27] |
| Ekstrand 2011b | 50 | 182126 | 25 | 64349 | 0.71 | [0.44; 1.14] |
| Random effects model | | | | | **0.62** | **[0.44; 0.89]** |
| Heterogeneity: $I^2 = 0\%$, $\tau^2 = 0$ | | | | | | |
| **Lower body** | | | | | | |
| Almutawa 2014 | 60 | 1901 | 64 | 1481 | 0.73 | [0.51; 1.04] |
| Bjørneboe 2010 | 227 | 74612 | 644 | 186929 | 0.88 | [0.76; 1.03] |
| Calloway 2019 | 511 | 345510 | 1558 | 1090980 | 1.04 | [0.94; 1.14] |
| Ekstrand 2006 | 243 | 58512 | 155 | 24362 | 0.65 | [0.53; 0.80] |
| Ekstrand 2011b | 866 | 182126 | 395 | 64349 | 0.77 | [0.69; 0.87] |
| Ekstrand 2012 | 10 | 274305 | 41 | 905219 | 0.80 | [0.40; 1.61] |
| Hägglund 2011a | 36 | 269550 | 101 | 906386 | 1.20 | [0.82; 1.75] |
| Kristenson 2013 | 217 | 156381 | 404 | 161655 | 0.56 | [0.47; 0.65] |
| Random effects model | | | | | **0.80** | **[0.67; 0.96]** |
| Heterogeneity: $I^2 = 87\%$, $\tau^2 = 0.0497$ | | | | | | |
| **Pelvis and thigh** | | | | | | |
| Almutawa 2014 | 14 | 1901 | 21 | 1481 | 0.52 | [0.26; 1.02] |
| Bjørneboe 2010 | 87 | 74612 | 250 | 186929 | 0.87 | [0.68; 1.11] |
| Calloway 2019 | 262 | 345510 | 796 | 1090980 | 1.04 | [0.90; 1.20] |
| Ekstrand 2006 | 40 | 58512 | 37 | 24362 | 0.45 | [0.29; 0.70] |
| Ekstrand 2011b | 339 | 182126 | 278 | 64349 | 0.43 | [0.37; 0.50] |
| Kristenson 2013 | 103 | 156381 | 197 | 161655 | 0.54 | [0.43; 0.69] |
| Random effects model | | | | | **0.62** | **[0.45; 0.85]** |
| Heterogeneity: $I^2 = 94\%$, $\tau^2 = 0.1311$ | | | | | | |
| **Knee** | | | | | | |
| Almutawa 2014 | 7 | 1901 | 19 | 1481 | 0.29 | [0.12; 0.68] |
| Bjørneboe 2010 | 53 | 74612 | 135 | 186929 | 0.98 | [0.72; 1.35] |
| Calloway 2019 | 70 | 345510 | 208 | 1090980 | 1.06 | [0.81; 1.39] |
| Ekstrand 2006 | 31 | 58512 | 22 | 24362 | 0.59 | [0.34; 1.01] |
| Ekstrand 2011b | 184 | 182126 | 116 | 64349 | 0.56 | [0.44; 0.71] |
| Hägglund 2011a | 36 | 269550 | 101 | 906386 | 1.20 | [0.82; 1.75] |
| Kristenson 2013 | 42 | 156381 | 87 | 161655 | 0.50 | [0.35; 0.72] |
| Random effects model | | | | | **0.71** | **[0.52; 0.99]** |
| Heterogeneity: $I^2 = 81\%$, $\tau^2 = 0.1476$ | | | | | | |
| **ACL** | | | | | | |
| Bjørneboe 2010 | 3 | 74612 | 11 | 186929 | 0.68 | [0.19; 2.45] |
| Calloway 2019 | 9 | 345510 | 30 | 1090980 | 0.95 | [0.45; 2.00] |
| Ekstrand 2011b | 0 | 182126 | 0 | 64349 | 0.35 | [0.01; 17.81] |
| Random effects model | | | | | **0.85** | **[0.45; 1.61]** |
| Heterogeneity: $I^2 = 0\%$, $\tau^2 = 0$ | | | | | | |
| **Foot and ankle** | | | | | | |
| Almutawa 2014 | 27 | 1901 | 13 | 1481 | 1.62 | [0.83; 3.14] |
| Bjørneboe 2010 | 87 | 74612 | 259 | 186929 | 0.84 | [0.66; 1.07] |
| Calloway 2019 | 179 | 345510 | 554 | 1090980 | 1.02 | [0.86; 1.21] |
| Ekstrand 2011b | 351 | 182126 | 216 | 64349 | 0.57 | [0.48; 0.68] |
| Kristenson 2013 | 72 | 156381 | 120 | 161655 | 0.62 | [0.46; 0.83] |
| Random effects model | | | | | **0.81** | **[0.60; 1.10]** |
| Heterogeneity: $I^2 = 87\%$, $\tau^2 = 0.0936$ | | | | | | |
| Heterogeneity: $I^2 = 84\%$, $\tau^2 = 0.0864$ | | | | | | |
| Test for subgroup differences: $\chi^2_6 = 4.29$, df = 6 ($p = 0.64$) | | | | | | |

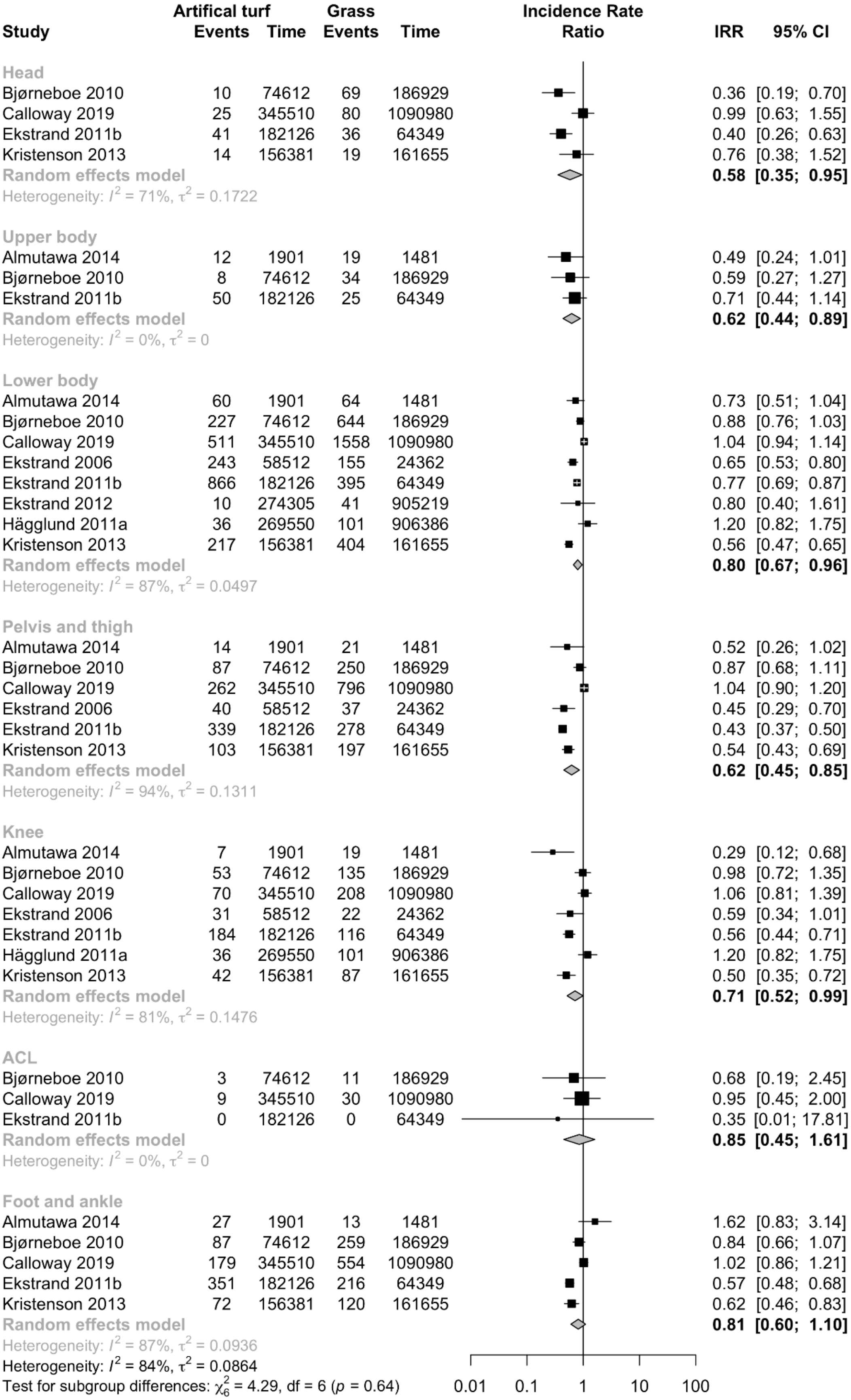

Supplementary figure 5: Forest plot of the injury incidence rate ratios on artificial turf compared to grass and other playing surfaces stratified by the anatomical location of the injury in amateur players.

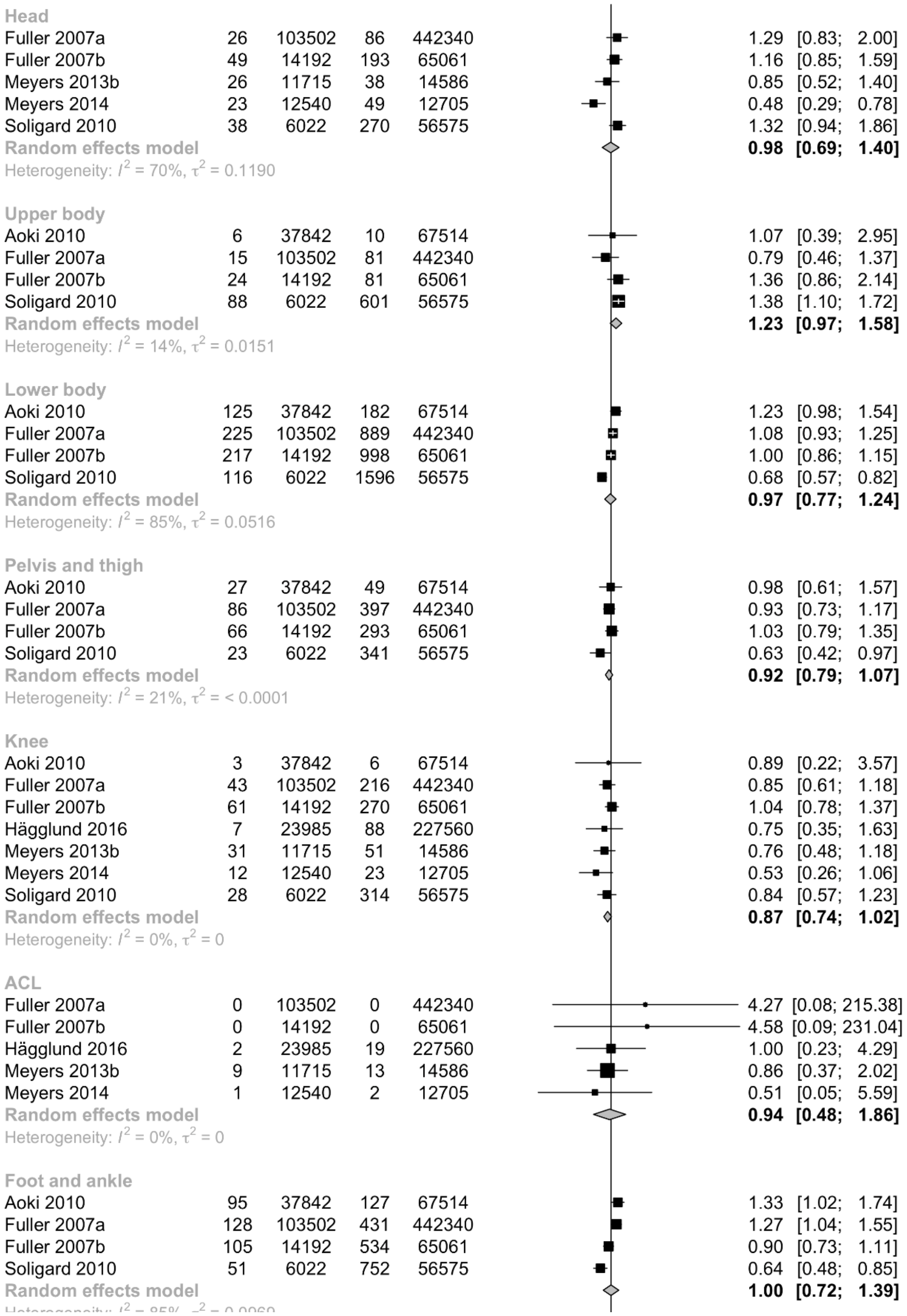

Supplementary figure 6: Forest plot of the injury incidence rate ratios on artificial turf compared to grass and other playing surfaces stratified by the anatomical location of the injury sustained in games.

| Study | Artifical turf Events | Artifical turf Time | Grass Events | Grass Time | IRR | 95% CI |
|---|---|---|---|---|---|---|
| **Head** | | | | | | |
| Calloway 2019 | 25 | 345510 | 80 | 1090980 | 0.99 | [0.63; 1.55] |
| Fuller 2007b | 49 | 14192 | 193 | 65061 | 1.16 | [0.85; 1.59] |
| Meyers 2013b | 26 | 11715 | 38 | 14586 | 0.85 | [0.52; 1.40] |
| Meyers 2014 | 23 | 12540 | 49 | 12705 | 0.48 | [0.29; 0.78] |
| Soligard 2010 | 38 | 6022 | 270 | 56575 | 1.32 | [0.94; 1.86] |
| **Random effects model** | | | | | **0.94** | **[0.67; 1.31]** |
| Heterogeneity: $I^2 = 68\%$, $\tau^2 = 0.1009$ | | | | | | |
| **Upper body** | | | | | | |
| Fuller 2007b | 24 | 14192 | 81 | 65061 | 1.36 | [0.86; 2.14] |
| Soligard 2010 | 88 | 6022 | 601 | 56575 | 1.38 | [1.10; 1.72] |
| **Random effects model** | | | | | **1.37** | **[1.12; 1.68]** |
| Heterogeneity: $I^2 = 0\%$, $\tau^2 = 0$ | | | | | | |
| **Lower body** | | | | | | |
| Calloway 2019 | 511 | 345510 | 1558 | 1090980 | 1.04 | [0.94; 1.14] |
| Fuller 2007b | 217 | 14192 | 998 | 65061 | 1.00 | [0.86; 1.15] |
| Soligard 2010 | 116 | 6022 | 1596 | 56575 | 0.68 | [0.57; 0.82] |
| **Random effects model** | | | | | **0.90** | **[0.70; 1.16]** |
| Heterogeneity: $I^2 = 87\%$, $\tau^2 = 0.0441$ | | | | | | |
| **Pelvis and thigh** | | | | | | |
| Calloway 2019 | 262 | 345510 | 796 | 1090980 | 1.04 | [0.90; 1.20] |
| Fuller 2007b | 66 | 14192 | 293 | 65061 | 1.03 | [0.79; 1.35] |
| Soligard 2010 | 23 | 6022 | 341 | 56575 | 0.63 | [0.42; 0.97] |
| **Random effects model** | | | | | **0.94** | **[0.73; 1.20]** |
| Heterogeneity: $I^2 = 59\%$, $\tau^2 = 0.0293$ | | | | | | |
| **Knee** | | | | | | |
| Calloway 2019 | 70 | 345510 | 208 | 1090980 | 1.06 | [0.81; 1.39] |
| Fuller 2007b | 61 | 14192 | 270 | 65061 | 1.04 | [0.78; 1.37] |
| Meyers 2013b | 31 | 11715 | 51 | 14586 | 0.76 | [0.48; 1.18] |
| Meyers 2014 | 12 | 12540 | 23 | 12705 | 0.53 | [0.26; 1.06] |
| Soligard 2010 | 28 | 6022 | 314 | 56575 | 0.84 | [0.57; 1.23] |
| **Random effects model** | | | | | **0.94** | **[0.80; 1.10]** |
| Heterogeneity: $I^2 = 22\%$, $\tau^2 = < 0.0001$ | | | | | | |
| **ACL** | | | | | | |
| Calloway 2019 | 9 | 345510 | 30 | 1090980 | 0.95 | [0.45; 2.00] |
| Fuller 2007b | 0 | 14192 | 0 | 65061 | 4.58 | [0.09; 231.04] |
| Meyers 2013b | 9 | 11715 | 13 | 14586 | 0.86 | [0.37; 2.02] |
| Meyers 2014 | 1 | 12540 | 2 | 12705 | 0.51 | [0.05; 5.59] |
| **Random effects model** | | | | | **0.91** | **[0.53; 1.56]** |
| Heterogeneity: $I^2 = 0\%$, $\tau^2 = 0$ | | | | | | |
| **Foot and ankle** | | | | | | |
| Calloway 2019 | 179 | 345510 | 554 | 1090980 | 1.02 | [0.86; 1.21] |
| Fuller 2007b | 105 | 14192 | 534 | 65061 | 0.90 | [0.73; 1.11] |
| Soligard 2010 | 51 | 6022 | 752 | 56575 | 0.64 | [0.48; 0.85] |
| **Random effects model** | | | | | **0.85** | **[0.66; 1.11]** |
| Heterogeneity: $I^2 = 74\%$, $\tau^2 = 0.0408$ | | | | | | |

Heterogeneity: $I^2 = 60\%$, $\tau^2 = 0.0344$

Test for subgroup differences: $\chi^2_6 = 12.81$, df = 6 ($p = 0.05$)

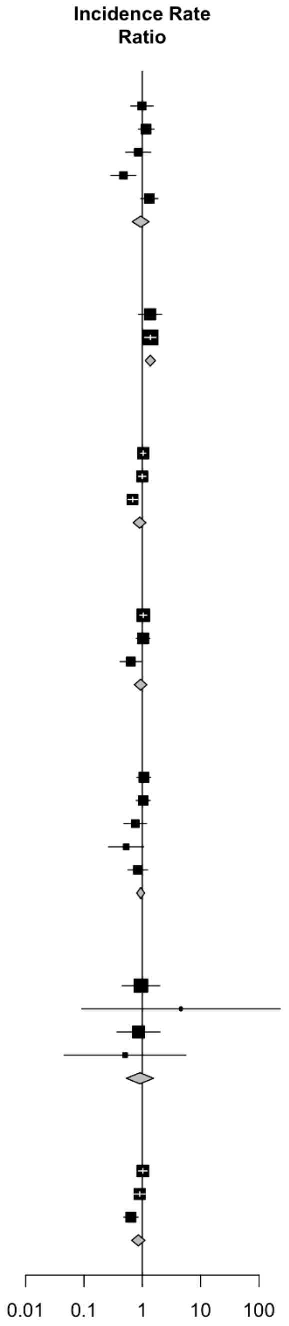

Supplementary figure 7: Forest plot of the injury incidence rate ratios on artificial turf compared to grass and other playing surfaces stratified by the anatomical location of the injury sustained in training sessions.

| Study | Artifical turf Events | Artifical turf Time | Grass Events | Grass Time | IRR | 95% CI |
|---|---|---|---|---|---|---|
| **Head** | | | | | | |
| Fuller 2007a | 26 | 103502 | 86 | 442340 | 1.29 | [0.83; 2.00] |
| **Upper body** | | | | | | |
| Fuller 2007a | 15 | 103502 | 81 | 442340 | 0.79 | [0.46; 1.37] |
| **Lower body** | | | | | | |
| Fuller 2007a | 225 | 103502 | 889 | 442340 | 1.08 | [0.93; 1.25] |
| **Pelvis and thigh** | | | | | | |
| Fuller 2007a | 86 | 103502 | 397 | 442340 | 0.93 | [0.73; 1.17] |
| **Knee** | | | | | | |
| Fuller 2007a | 43 | 103502 | 216 | 442340 | 0.85 | [0.61; 1.18] |
| **ACL** | | | | | | |
| Fuller 2007a | 0 | 103502 | 0 | 442340 | 4.27 | [0.08; 215.38] |
| **Foot and ankle** | | | | | | |
| Fuller 2007a | 128 | 103502 | 431 | 442340 | 1.27 | [1.04; 1.55] |

Heterogeneity: $I^2 = 30\%$, $\tau^2 = 0.0109$

Test for subgroup differences: $\chi^2_6 = 8.60$, df = 6 ($p$ = 0.20)

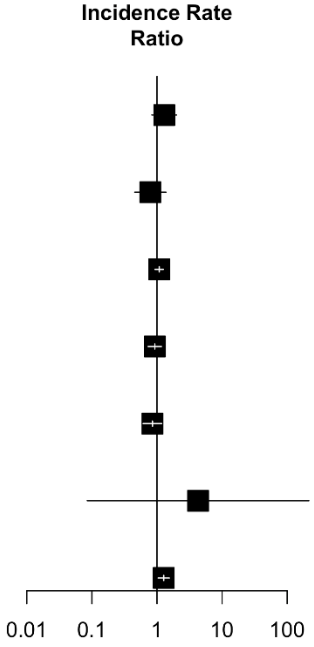

Supplementary figure 8: Forest plot of the injury incidence rate ratios on artificial turf compared to grass and other playing surfaces stratified by the anatomical location of the injury in adult players.

| Study | Artifical turf Events | Artifical turf Time | Grass Events | Grass Time | IRR | 95% CI |
|---|---|---|---|---|---|---|
| **Head** | | | | | | |
| Bjørneboe 2010 | 10 | 74612 | 69 | 186929 | 0.36 | [0.19; 0.70] |
| Calloway 2019 | 25 | 345510 | 80 | 1090980 | 0.99 | [0.63; 1.55] |
| Ekstrand 2011b | 41 | 182126 | 36 | 64349 | 0.40 | [0.26; 0.63] |
| Fuller 2007a | 26 | 103502 | 86 | 442340 | 1.29 | [0.83; 2.00] |
| Fuller 2007b | 49 | 14192 | 193 | 65061 | 1.16 | [0.85; 1.59] |
| Kristenson 2013 | 14 | 156381 | 19 | 161655 | 0.76 | [0.38; 1.52] |
| Meyers 2013b | 26 | 11715 | 38 | 14586 | 0.85 | [0.52; 1.40] |
| Meyers 2014 | 23 | 12540 | 49 | 12705 | 0.48 | [0.29; 0.78] |
| Random effects model | | | | | **0.73** | **[0.52; 1.03]** |
| Heterogeneity: $I^2 = 76\%$, $\tau^2 = 0.1810$ | | | | | | |
| **Upper body** | | | | | | |
| Almutawa 2014 | 12 | 1901 | 19 | 1481 | 0.49 | [0.24; 1.01] |
| Bjørneboe 2010 | 8 | 74612 | 34 | 186929 | 0.59 | [0.27; 1.27] |
| Ekstrand 2011b | 50 | 182126 | 25 | 64349 | 0.71 | [0.44; 1.14] |
| Fuller 2007a | 15 | 103502 | 81 | 442340 | 0.79 | [0.46; 1.37] |
| Fuller 2007b | 24 | 14192 | 81 | 65061 | 1.36 | [0.86; 2.14] |
| Random effects model | | | | | **0.79** | **[0.55; 1.12]** |
| Heterogeneity: $I^2 = 48\%$, $\tau^2 = 0.0795$ | | | | | | |
| **Lower body** | | | | | | |
| Almutawa 2014 | 60 | 1901 | 64 | 1481 | 0.73 | [0.51; 1.04] |
| Bjørneboe 2010 | 227 | 74612 | 644 | 186929 | 0.88 | [0.76; 1.03] |
| Calloway 2019 | 511 | 345510 | 1558 | 1090980 | 1.04 | [0.94; 1.14] |
| Ekstrand 2006 | 243 | 58512 | 155 | 24362 | 0.65 | [0.53; 0.80] |
| Ekstrand 2011b | 866 | 182126 | 395 | 64349 | 0.77 | [0.69; 0.87] |
| Ekstrand 2012 | 10 | 274305 | 41 | 905219 | 0.80 | [0.40; 1.61] |
| Fuller 2007a | 225 | 103502 | 889 | 442340 | 1.08 | [0.93; 1.25] |
| Fuller 2007b | 217 | 14192 | 998 | 65061 | 1.00 | [0.86; 1.15] |
| Hägglund 2011a | 36 | 269550 | 101 | 906386 | 1.20 | [0.82; 1.75] |
| Kristenson 2013 | 217 | 156381 | 404 | 161655 | 0.56 | [0.47; 0.65] |
| Random effects model | | | | | **0.85** | **[0.73; 0.99]** |
| Heterogeneity: $I^2 = 87\%$, $\tau^2 = 0.0499$ | | | | | | |
| **Pelvis and thigh** | | | | | | |
| Almutawa 2014 | 14 | 1901 | 21 | 1481 | 0.52 | [0.26; 1.02] |
| Bjørneboe 2010 | 87 | 74612 | 250 | 186929 | 0.87 | [0.68; 1.11] |
| Calloway 2019 | 262 | 345510 | 796 | 1090980 | 1.04 | [0.90; 1.20] |
| Ekstrand 2006 | 40 | 58512 | 37 | 24362 | 0.45 | [0.29; 0.70] |
| Ekstrand 2011b | 339 | 182126 | 278 | 64349 | 0.43 | [0.37; 0.50] |
| Fuller 2007a | 86 | 103502 | 397 | 442340 | 0.93 | [0.73; 1.17] |
| Fuller 2007b | 66 | 14192 | 293 | 65061 | 1.03 | [0.79; 1.35] |
| Kristenson 2013 | 103 | 156381 | 197 | 161655 | 0.54 | [0.43; 0.69] |
| Random effects model | | | | | **0.70** | **[0.53; 0.92]** |
| Heterogeneity: $I^2 = 92\%$, $\tau^2 = 0.1285$ | | | | | | |
| **Knee** | | | | | | |
| Almutawa 2014 | 7 | 1901 | 19 | 1481 | 0.29 | [0.12; 0.68] |
| Bjørneboe 2010 | 53 | 74612 | 135 | 186929 | 0.98 | [0.72; 1.35] |
| Calloway 2019 | 70 | 345510 | 208 | 1090980 | 1.06 | [0.81; 1.39] |
| Ekstrand 2006 | 31 | 58512 | 22 | 24362 | 0.59 | [0.34; 1.01] |
| Ekstrand 2011b | 184 | 182126 | 116 | 64349 | 0.56 | [0.44; 0.71] |
| Fuller 2007a | 43 | 103502 | 216 | 442340 | 0.85 | [0.61; 1.18] |
| Fuller 2007b | 61 | 14192 | 270 | 65061 | 1.04 | [0.78; 1.37] |
| Hägglund 2011a | 36 | 269550 | 101 | 906386 | 1.20 | [0.82; 1.75] |
| Kristenson 2013 | 42 | 156381 | 87 | 161655 | 0.50 | [0.35; 0.72] |
| Meyers 2013b | 31 | 11715 | 51 | 14586 | 0.76 | [0.48; 1.18] |
| Meyers 2014 | 12 | 12540 | 23 | 12705 | 0.53 | [0.26; 1.06] |
| Random effects model | | | | | **0.76** | **[0.61; 0.94]** |
| Heterogeneity: $I^2 = 73\%$, $\tau^2 = 0.0877$ | | | | | | |
| **ACL** | | | | | | |
| Bjørneboe 2010 | 3 | 74612 | 11 | 186929 | 0.68 | [0.19; 2.45] |
| Calloway 2019 | 9 | 345510 | 30 | 1090980 | 0.95 | [0.45; 2.00] |
| Ekstrand 2011b | 0 | 182126 | 0 | 64349 | 0.35 | [0.01; 17.81] |
| Fuller 2007a | 0 | 103502 | 0 | 442340 | 4.27 | [0.08; 215.38] |
| Fuller 2007b | 0 | 14192 | 0 | 65061 | 4.58 | [0.09; 231.04] |
| Meyers 2013b | 9 | 11715 | 13 | 14586 | 0.86 | [0.37; 2.02] |
| Meyers 2014 | 1 | 12540 | 2 | 12705 | 0.51 | [0.05; 5.59] |
| Random effects model | | | | | **0.88** | **[0.54; 1.44]** |
| Heterogeneity: $I^2 = 0\%$, $\tau^2 = 0$ | | | | | | |
| **Foot and ankle** | | | | | | |
| Almutawa 2014 | 27 | 1901 | 13 | 1481 | 1.62 | [0.83; 3.14] |
| Bjørneboe 2010 | 87 | 74612 | 259 | 186929 | 0.84 | [0.66; 1.07] |
| Calloway 2019 | 179 | 345510 | 554 | 1090980 | 1.02 | [0.86; 1.21] |
| Ekstrand 2011b | 351 | 182126 | 216 | 64349 | 0.57 | [0.48; 0.68] |
| Fuller 2007a | 128 | 103502 | 431 | 442340 | 1.27 | [1.04; 1.55] |
| Fuller 2007b | 105 | 14192 | 534 | 65061 | 0.90 | [0.73; 1.11] |
| Kristenson 2013 | 72 | 156381 | 120 | 161655 | 0.62 | [0.46; 0.83] |
| Random effects model | | | | | **0.89** | **[0.69; 1.13]** |
| Heterogeneity: $I^2 = 88\%$, $\tau^2 = 0.0893$ | | | | | | |

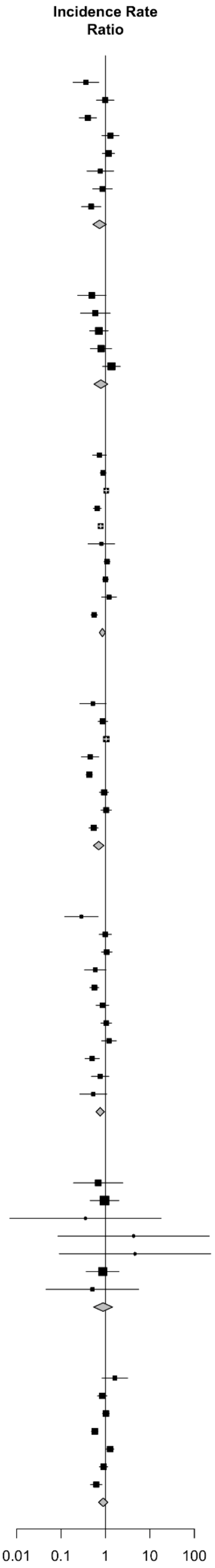


Heterogeneity: $I^2 = 82\%$, $\tau^2 = 0.0831$

Test for subgroup differences: $\chi^2_6 = 2.79$, df = 6 ($p = 0.83$)

Supplementary figure 9: Forest plot of the injury incidence rate ratios on artificial turf compared to grass and other playing surfaces stratified by the anatomical location of the injury in youth players.

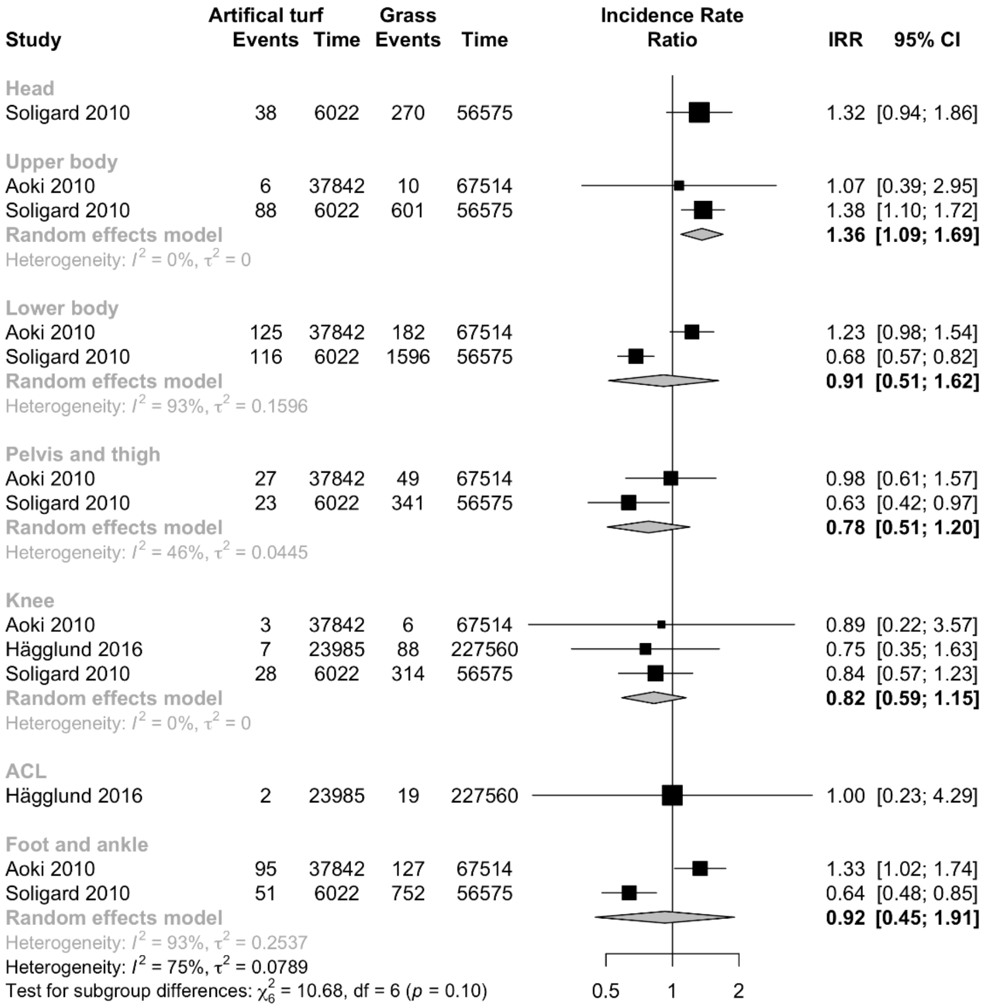

Supplementary figure 10: Forest plot of the injury incidence rate ratios on artificial turf compared to grass stratified by the geographical global regions.

| Study | Artifical turf Events | Artifical turf Time | Grass Events | Grass Time | IRR | 95%-CI |
|---|---|---|---|---|---|---|
| **Middle-East** | | | | | | |
| Almutawa 2014 | 72 | 1901 | 83 | 1481 | 0.68 | [0.49; 0.93] |
| **Central Europe, East-Asia, and the USA** | | | | | | |
| Aoki 2010 | 186 | 37842 | 298 | 67514 | 1.11 | [0.93; 1.34] |
| Calloway 2019 | 536 | 345510 | 1638 | 1090980 | 1.03 | [0.94; 1.14] |
| Ekstrand 2006 | 483 | 58512 | 274 | 24362 | 0.73 | [0.63; 0.85] |
| Ekstrand 2011b | 998 | 182126 | 494 | 64349 | 0.71 | [0.64; 0.80] |
| Fuller 2007a | 311 | 103502 | 1283 | 442340 | 1.04 | [0.92; 1.17] |
| Fuller 2007b | 317 | 14192 | 1477 | 65061 | 0.98 | [0.87; 1.11] |
| Lanzetti 2017 | 23 | 1270 | 20 | 1310 | 1.19 | [0.65; 2.16] |
| Meyers 2013b | 272 | 11715 | 421 | 14586 | 0.80 | [0.69; 0.94] |
| Meyers 2014 | 268 | 12540 | 454 | 12705 | 0.60 | [0.51; 0.70] |
| Rössler 2017 | 166 | 115711 | 161 | 156011 | 1.39 | [1.12; 1.73] |
| **Random effects model** | | | | | **0.91** | **[0.78; 1.07]** |
| Heterogeneity: $I^2 = 90\%$, $\tau^2 = 0.0582$ | | | | | | |
| **Northern Europe** | | | | | | |
| Bjørneboe 2010 | 267 | 74612 | 800 | 186929 | 0.84 | [0.73; 0.96] |
| Kristenson 2013 | 375 | 156381 | 591 | 161655 | 0.66 | [0.58; 0.75] |
| Kristenson 2016 | 119 | 5938 | 253 | 10513 | 0.83 | [0.67; 1.04] |
| Soligard 2010 | 206 | 6022 | 2248 | 56575 | 0.86 | [0.75; 0.99] |
| Steffen 2007 | 119 | 39979 | 286 | 73044 | 0.76 | [0.61; 0.94] |
| **Random effects model** | | | | | **0.78** | **[0.70; 0.87]** |
| Heterogeneity: $I^2 = 60\%$, $\tau^2 = 0.0093$ | | | | | | |

Heterogeneity: $I^2 = 87\%$, $\tau^2 = 0.0420$

Test for subgroup differences: $\chi^2_2 = 3.74$, df = 2 ($p = 0.15$)

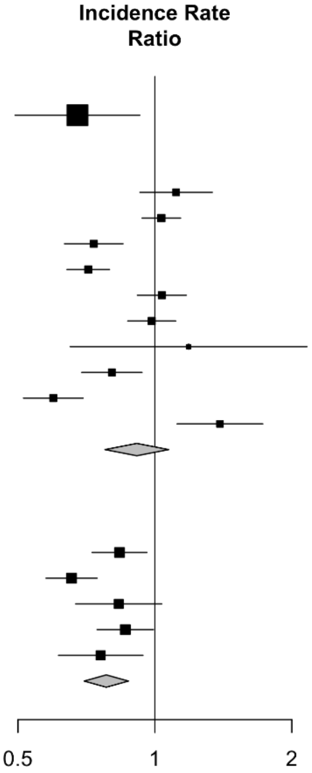

Supplementary figure 11: Sensitivity analysis with only third generation artificial turfs included. Forest plot of the incidence of overall injuries on artificial turf compared to grass stratified by sex.

| Study | Artifical turf Events | Artifical turf Time | Grass Events | Grass Time | IRR | 95%-CI |
|---|---|---|---|---|---|---|
| **Both** | | | | | | |
| Soligard 2010 | 206 | 6022 | 2248 | 56575 | 0.86 | [0.75; 0.99] |
| **Men** | | | | | | |
| Almutawa 2014 | 72 | 1901 | 83 | 1481 | 0.68 | [0.49; 0.93] |
| Bjørneboe 2010 | 267 | 74612 | 800 | 186929 | 0.84 | [0.73; 0.96] |
| Calloway 2019 | 536 | 345510 | 1638 | 1090980 | 1.03 | [0.94; 1.14] |
| Ekstrand 2006 | 483 | 58512 | 274 | 24362 | 0.73 | [0.63; 0.85] |
| Ekstrand 2011b | 832 | 143571 | 437 | 54500 | 0.72 | [0.64; 0.81] |
| Fuller 2007a | 189 | 56504 | 629 | 208842 | 1.11 | [0.94; 1.31] |
| Fuller 2007b | 183 | 7195 | 665 | 27803 | 1.06 | [0.90; 1.25] |
| Kristenson 2013 | 375 | 156381 | 591 | 161655 | 0.66 | [0.58; 0.75] |
| Kristenson 2016 | 119 | 5938 | 253 | 10513 | 0.83 | [0.67; 1.04] |
| Lanzetti 2017 | 23 | 1270 | 20 | 1310 | 1.19 | [0.65; 2.16] |
| Meyers 2014 | 268 | 12540 | 454 | 12705 | 0.60 | [0.51; 0.70] |
| **Random effects model** | | | | | **0.82** | **[0.72; 0.94]** |
| Heterogeneity: $I^2 = 88\%$, $\tau^2 = 0.0417$ | | | | | | |
| **Women** | | | | | | |
| Ekstrand 2011b | 166 | 38555 | 57 | 9849 | 0.74 | [0.55; 1.01] |
| Fuller 2007a | 122 | 46998 | 654 | 233498 | 0.93 | [0.76; 1.12] |
| Fuller 2007b | 134 | 6997 | 812 | 37258 | 0.88 | [0.73; 1.05] |
| Meyers 2013b | 272 | 11715 | 421 | 14586 | 0.80 | [0.69; 0.94] |
| Steffen 2007 | 119 | 39979 | 286 | 73044 | 0.76 | [0.61; 0.94] |
| **Random effects model** | | | | | **0.83** | **[0.76; 0.91]** |
| Heterogeneity: $I^2 = 0\%$, $\tau^2 = 0$ | | | | | | |

Heterogeneity: $I^2 = 81\%$, $\tau^2 = 0.0258$

Test for subgroup differences: $\chi^2_2 = 0.23$, df = 2 ($p = 0.89$)

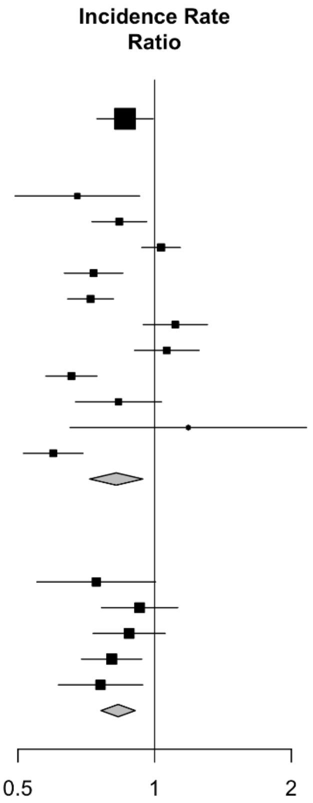

Supplementary figure 12: Sensitivity analysis with only third generation artificial turfs included. Forest plot of the injury incidence rate ratios on artificial turf compared to grass and other playing surfaces stratified between professional and amateur players.

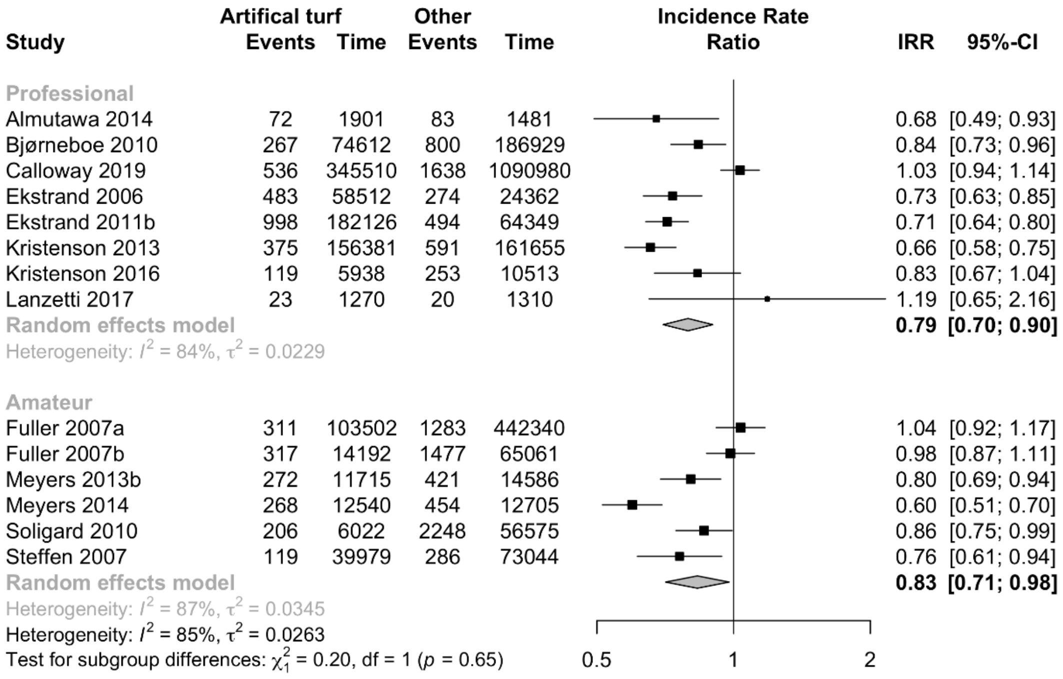

Supplementary figure 13: Sensitivity analysis with only third generation artificial turfs included. Forest plot of the injury incidence rate ratios on artificial turf compared to grass and other playing surfaces stratified by matches and training sessions.

| Study | Artifical turf Events | Artifical turf Time | Other Events | Other Time | IRR | 95%-CI |
|---|---|---|---|---|---|---|
| **Both** | | | | | | |
| Almutawa 2014 | 72 | 1901 | 83 | 1481 | 0.68 | [0.49; 0.93] |
| Bjørneboe 2010 | 267 | 74612 | 800 | 186929 | 0.84 | [0.73; 0.96] |
| Ekstrand 2006 | 483 | 58512 | 274 | 24362 | 0.73 | [0.63; 0.85] |
| Ekstrand 2011b | 998 | 182126 | 494 | 64349 | 0.71 | [0.64; 0.80] |
| Kristenson 2013 | 375 | 156381 | 591 | 161655 | 0.66 | [0.58; 0.75] |
| Kristenson 2016 | 119 | 5938 | 253 | 10513 | 0.83 | [0.67; 1.04] |
| Steffen 2007 | 119 | 39979 | 286 | 73044 | 0.76 | [0.61; 0.94] |
| **Random effects model** | | | | | **0.74** | **[0.69; 0.80]** |
| Heterogeneity: $I^2 = 27\%$, $\tau^2 = 0.0032$ | | | | | | |
| **Match** | | | | | | |
| Calloway 2019 | 536 | 345510 | 1638 | 1090980 | 1.03 | [0.94; 1.14] |
| Fuller 2007b | 317 | 14192 | 1477 | 65061 | 0.98 | [0.87; 1.11] |
| Lanzetti 2017 | 23 | 1270 | 20 | 1310 | 1.19 | [0.65; 2.16] |
| Meyers 2013b | 272 | 11715 | 421 | 14586 | 0.80 | [0.69; 0.94] |
| Meyers 2014 | 268 | 12540 | 454 | 12705 | 0.60 | [0.51; 0.70] |
| Soligard 2010 | 206 | 6022 | 2248 | 56575 | 0.86 | [0.75; 0.99] |
| **Random effects model** | | | | | **0.86** | **[0.72; 1.03]** |
| Heterogeneity: $I^2 = 88\%$, $\tau^2 = 0.0404$ | | | | | | |
| **Training** | | | | | | |
| Fuller 2007a | 311 | 103502 | 1283 | 442340 | 1.04 | [0.92; 1.17] |

Heterogeneity: $I^2 = 85\%$, $\tau^2 = 0.0263$

Test for subgroup differences: $\chi^2_2 = 21.50$, df = 2 ($p < 0.01$)

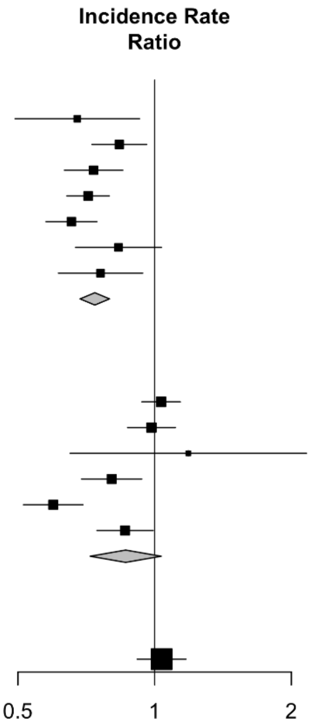

Supplementary figure 14: Sensitivity analysis with only third generation artificial turfs included. Forest plot of the injury incidence rate ratios on artificial turf compared to grass and other playing surfaces stratified by injury mechanism (contact vs non-contact).

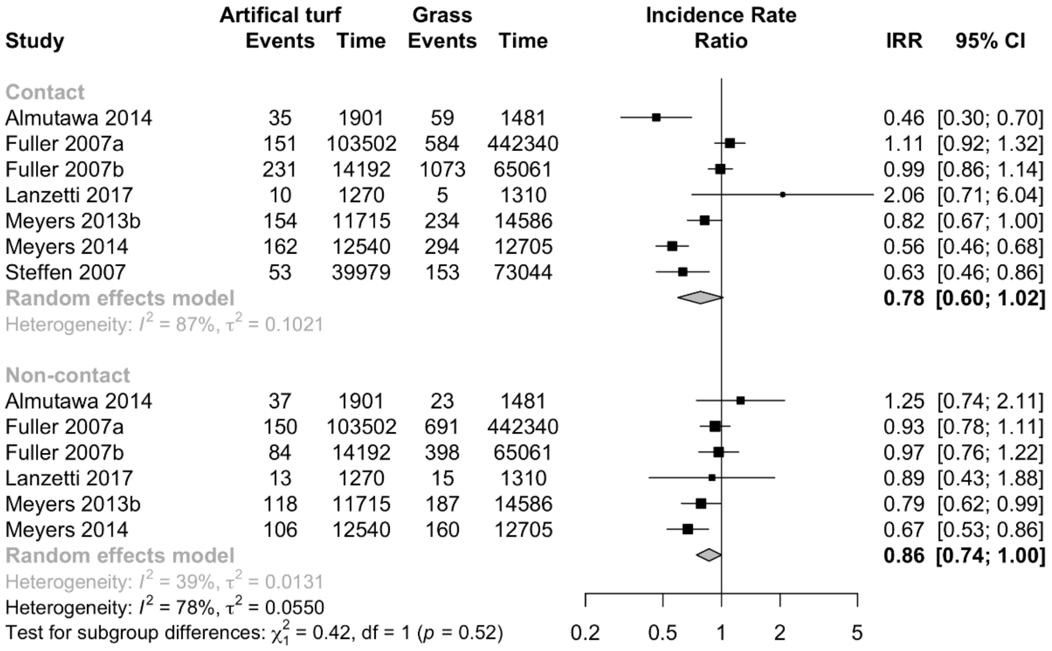

Supplementary figure 15: Sensitivity analysis with only third generation artificial turfs included. Forest plot of the injury incidence rate ratios on artificial turf compared to grass and other playing surfaces stratified by injury type (fracture, sprain, ligament injury).

| Study | Artifical turf Events | Artifical turf Time | Grass Events | Grass Time | IRR | 95% CI |
|---|---|---|---|---|---|---|
| **Contusion** | | | | | | |
| Bjørneboe 2010 | 53 | 74612 | 153 | 186929 | 0.87 | [0.64; 1.19] |
| Fuller 2007a | 43 | 103502 | 165 | 442340 | 1.11 | [0.80; 1.56] |
| Fuller 2007b | 70 | 14192 | 364 | 65061 | 0.88 | [0.68; 1.14] |
| Kristenson 2013 | 97 | 156381 | 93 | 161655 | 1.08 | [0.81; 1.43] |
| Lanzetti 2017 | 6 | 1270 | 1 | 1310 | 6.19 | [0.75; 51.41] |
| Soligard 2010 | 83 | 6022 | 883 | 56575 | 0.88 | [0.71; 1.11] |
| **Random effects model** | | | | | **0.95** | **[0.84; 1.07]** |
| Heterogeneity: $I^2$ = 12%, $\tau^2$ = < 0.0001 | | | | | | |
| **Sprain** | | | | | | |
| Almutawa 2014 | 12 | 1901 | 20 | 1481 | 0.47 | [0.23; 0.96] |
| Bjørneboe 2010 | 100 | 74612 | 279 | 186929 | 0.90 | [0.71; 1.13] |
| Calloway 2019 | 118 | 345510 | 328 | 1090980 | 1.14 | [0.92; 1.40] |
| Ekstrand 2006 | 99 | 58512 | 46 | 24362 | 0.90 | [0.63; 1.27] |
| Fuller 2007a | 110 | 103502 | 440 | 442340 | 1.07 | [0.87; 1.32] |
| Fuller 2007b | 117 | 14192 | 547 | 65061 | 0.98 | [0.80; 1.20] |
| Kristenson 2013 | 119 | 156381 | 186 | 161655 | 0.66 | [0.53; 0.83] |
| Lanzetti 2017 | 0 | 1270 | 1 | 1310 | 0.34 | [0.01; 8.44] |
| Soligard 2010 | 6 | 6022 | 123 | 56575 | 0.46 | [0.20; 1.04] |
| **Random effects model** | | | | | **0.88** | **[0.74; 1.04]** |
| Heterogeneity: $I^2$ = 61%, $\tau^2$ = 0.0346 | | | | | | |
| **Strain** | | | | | | |
| Almutawa 2014 | 45 | 1901 | 49 | 1481 | 0.72 | [0.48; 1.07] |
| Bjørneboe 2010 | 88 | 74612 | 258 | 186929 | 0.85 | [0.67; 1.09] |
| Calloway 2019 | 285 | 345510 | 842 | 1090980 | 1.07 | [0.93; 1.22] |
| Ekstrand 2006 | 59 | 58512 | 61 | 24362 | 0.40 | [0.28; 0.58] |
| Ekstrand 2011a | 492 | 269550 | 2416 | 906386 | 0.68 | [0.62; 0.75] |
| Fuller 2007a | 120 | 103502 | 525 | 442340 | 0.98 | [0.80; 1.19] |
| Fuller 2007b | 66 | 14192 | 298 | 65061 | 1.02 | [0.78; 1.33] |
| Kristenson 2013 | 124 | 156381 | 251 | 161655 | 0.51 | [0.41; 0.63] |
| Lanzetti 2017 | 13 | 1270 | 14 | 1310 | 0.96 | [0.45; 2.04] |
| Soligard 2010 | 13 | 6022 | 168 | 56575 | 0.73 | [0.41; 1.28] |
| **Random effects model** | | | | | **0.76** | **[0.62; 0.93]** |
| Heterogeneity: $I^2$ = 86%, $\tau^2$ = 0.0835 | | | | | | |
| **Other** | | | | | | |
| Almutawa 2014 | 15 | 1901 | 14 | 1481 | 0.83 | [0.40; 1.73] |
| Bjørneboe 2010 | 26 | 74612 | 110 | 186929 | 0.59 | [0.39; 0.91] |
| Ekstrand 2012 | 10 | 274305 | 41 | 905219 | 0.80 | [0.40; 1.61] |
| Fuller 2007a | 68 | 103502 | 234 | 442340 | 1.24 | [0.95; 1.63] |
| Fuller 2007b | 64 | 14192 | 268 | 65061 | 1.09 | [0.83; 1.44] |
| Kristenson 2013 | 33 | 156381 | 55 | 161655 | 0.62 | [0.40; 0.95] |
| Lanzetti 2017 | 4 | 1270 | 4 | 1310 | 1.03 | [0.26; 4.12] |
| Soligard 2010 | 8 | 6022 | 89 | 56575 | 0.84 | [0.41; 1.74] |
| **Random effects model** | | | | | **0.87** | **[0.68; 1.12]** |
| Heterogeneity: $I^2$ = 49%, $\tau^2$ = 0.0594 | | | | | | |

Heterogeneity: $I^2$ = 74%, $\tau^2$ = 0.0514

Test for subgroup differences: $\chi^2_3$ = 3.34, df = 3 ($p$ = 0.34)

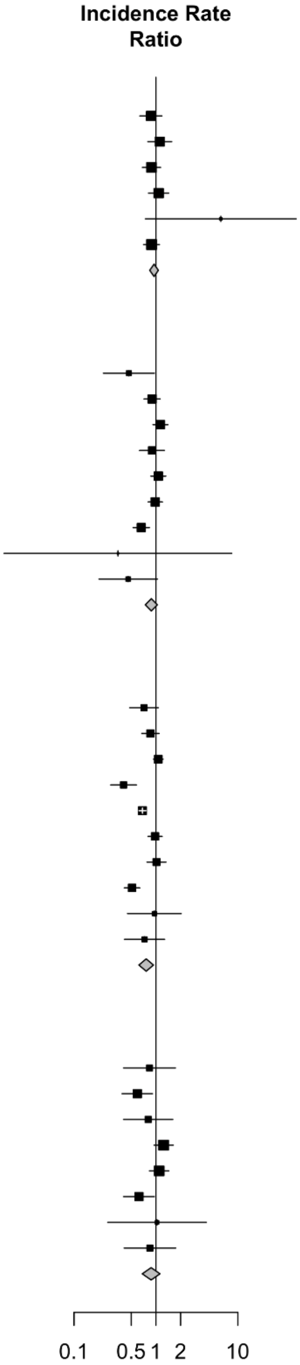

Supplementary figure 16: Sensitivity analysis with only prospective studies included. Forest plot of the incidence of overall injuries on artificial turf compared to grass stratified by sex.

| Study | Artifical turf Events | Artifical turf Time | Grass Events | Grass Time | IRR | 95%-CI |
|---|---|---|---|---|---|---|
| **Both** | | | | | | |
| Aoki 2010 | 186 | 37842 | 298 | 67514 | 1.11 | [0.93; 1.34] |
| Rössler 2017 | 166 | 115711 | 161 | 156011 | 1.39 | [1.12; 1.73] |
| Soligard 2010 | 206 | 6022 | 2248 | 56575 | 0.86 | [0.75; 0.99] |
| **Random effects model** | | | | | **1.09** | **[0.83; 1.43]** |
| Heterogeneity: $I^2 = 86\%$, $\tau^2 = 0.0492$ | | | | | | |
| **Men** | | | | | | |
| Almutawa 2014 | 72 | 1901 | 83 | 1481 | 0.68 | [0.49; 0.93] |
| Bjørneboe 2010 | 267 | 74612 | 800 | 186929 | 0.84 | [0.73; 0.96] |
| Ekstrand 2006 | 483 | 58512 | 274 | 24362 | 0.73 | [0.63; 0.85] |
| Ekstrand 2011b | 832 | 143571 | 437 | 54500 | 0.72 | [0.64; 0.81] |
| Fuller 2007a | 189 | 56504 | 629 | 208842 | 1.11 | [0.94; 1.31] |
| Fuller 2007b | 183 | 7195 | 665 | 27803 | 1.06 | [0.90; 1.25] |
| Kristenson 2013 | 375 | 156381 | 591 | 161655 | 0.66 | [0.58; 0.75] |
| Kristenson 2016 | 119 | 5938 | 253 | 10513 | 0.83 | [0.67; 1.04] |
| Lanzetti 2017 | 23 | 1270 | 20 | 1310 | 1.19 | [0.65; 2.16] |
| Meyers 2014 | 268 | 12540 | 454 | 12705 | 0.60 | [0.51; 0.70] |
| **Random effects model** | | | | | **0.80** | **[0.70; 0.92]** |
| Heterogeneity: $I^2 = 84\%$, $\tau^2 = 0.0391$ | | | | | | |
| **Women** | | | | | | |
| Ekstrand 2011b | 166 | 38555 | 57 | 9849 | 0.74 | [0.55; 1.01] |
| Fuller 2007a | 122 | 46998 | 654 | 233498 | 0.93 | [0.76; 1.12] |
| Fuller 2007b | 134 | 6997 | 812 | 37258 | 0.88 | [0.73; 1.05] |
| Meyers 2013b | 272 | 11715 | 421 | 14586 | 0.80 | [0.69; 0.94] |
| Steffen 2007 | 119 | 39979 | 286 | 73044 | 0.76 | [0.61; 0.94] |
| **Random effects model** | | | | | **0.83** | **[0.76; 0.91]** |
| Heterogeneity: $I^2 = 0\%$, $\tau^2 = 0$ | | | | | | |

Heterogeneity: $I^2 = 83\%$, $\tau^2 = 0.0385$

Test for subgroup differences: $\chi^2_2 = 4.04$, df = 2 ($p = 0.13$)

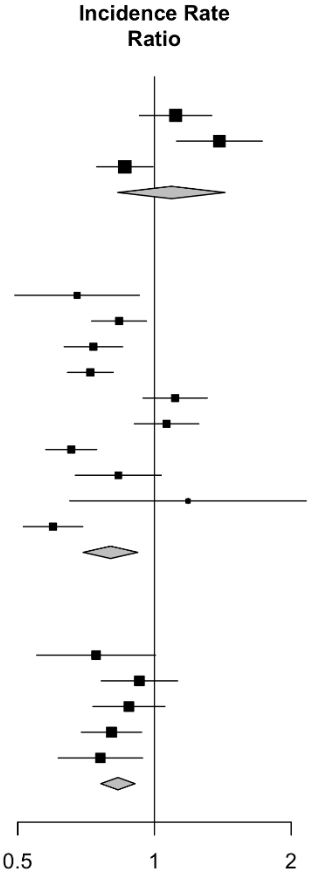

Supplementary figure 17: Sensitivity analysis with only prospective studies included. Forest plot of the injury incidence rate ratios on artificial turf compared to grass and other playing surfaces stratified between professional and amateur players.

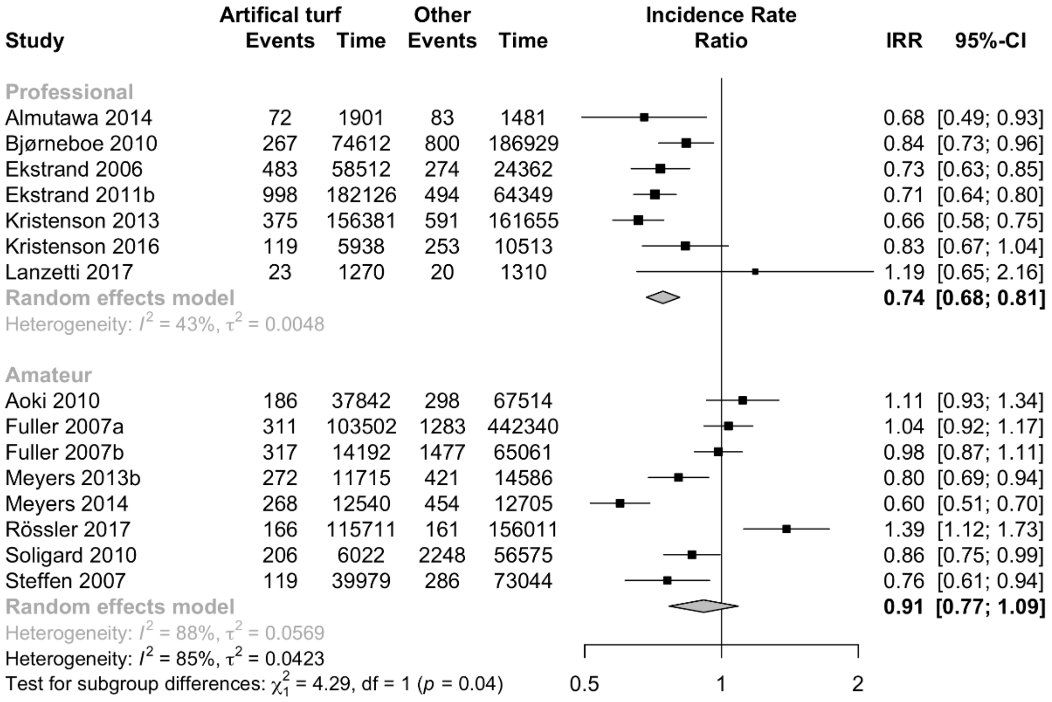

Supplementary figure 18: Sensitivity analysis with only prospective studies included. Forest plot of the injury incidence rate ratios on artificial turf compared to grass and other playing surfaces stratified by matches and training sessions.

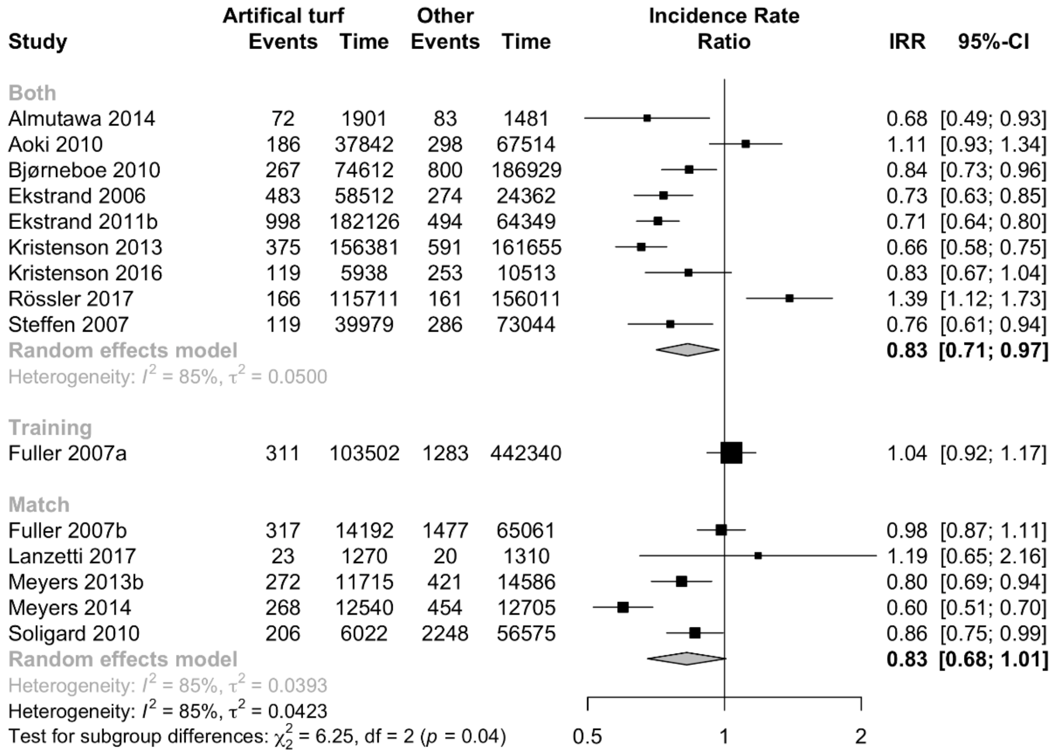

Supplementary figure 19: Sensitivity analysis with only prospective studies included. Forest plot of the injury incidence rate ratios on artificial turf compared to grass and other playing surfaces stratified by injury mechanism (contact vs non-contact).

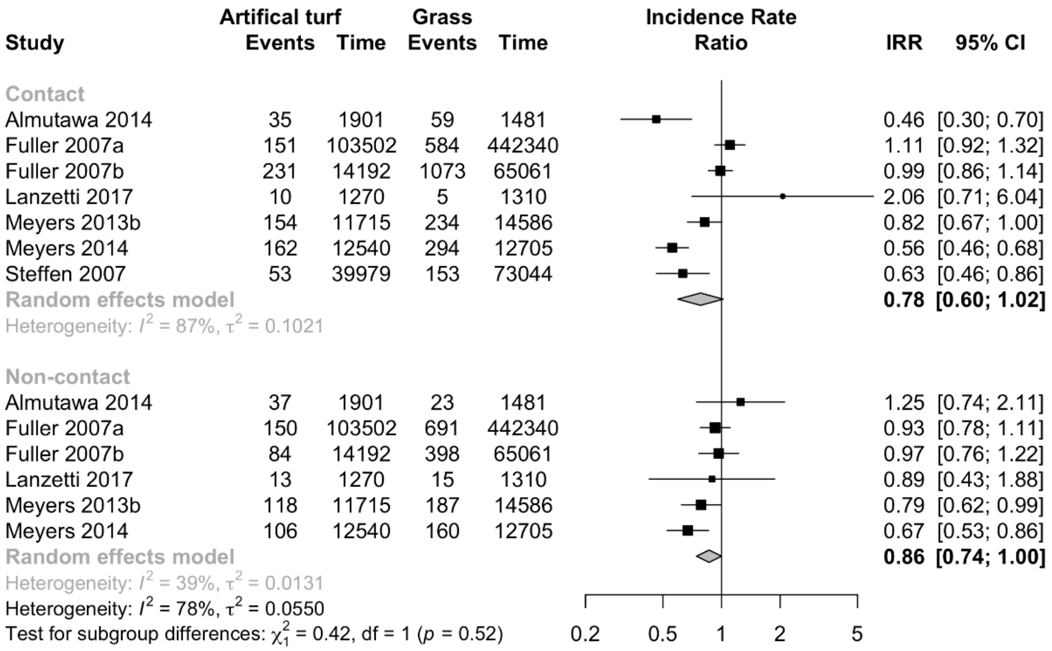

Supplementary figure 20: Sensitivity analysis with only prospective studies included. Forest plot of the injury incidence rate ratios on artificial turf compared to grass and other playing surfaces stratified by injury type (fracture, sprain, ligament injury).

| Study | Artifical turf Events | Artifical turf Time | Grass Events | Grass Time | IRR | 95% CI |
|---|---|---|---|---|---|---|
| **Contusion** | | | | | | |
| Aoki 2010 | 20 | 37842 | 30 | 67514 | 1.19 | [0.68; 2.09] |
| Bjørneboe 2010 | 53 | 74612 | 153 | 186929 | 0.87 | [0.64; 1.19] |
| Fuller 2007a | 43 | 103502 | 165 | 442340 | 1.11 | [0.80; 1.56] |
| Fuller 2007b | 70 | 14192 | 364 | 65061 | 0.88 | [0.68; 1.14] |
| Kristenson 2013 | 97 | 156381 | 93 | 161655 | 1.08 | [0.81; 1.43] |
| Lanzetti 2017 | 6 | 1270 | 1 | 1310 | 6.19 | [0.75; 51.41] |
| Soligard 2010 | 83 | 6022 | 883 | 56575 | 0.88 | [0.71; 1.11] |
| Random effects model | | | | | **0.96** | **[0.85; 1.08]** |
| Heterogeneity: $I^2 = 4\%$, $\tau^2 = < 0.0001$ | | | | | | |
| **Sprain** | | | | | | |
| Almutawa 2014 | 12 | 1901 | 20 | 1481 | 0.47 | [0.23; 0.96] |
| Aoki 2010 | 92 | 37842 | 133 | 67514 | 1.23 | [0.95; 1.61] |
| Bjørneboe 2010 | 100 | 74612 | 279 | 186929 | 0.90 | [0.71; 1.13] |
| Ekstrand 2006 | 99 | 58512 | 46 | 24362 | 0.90 | [0.63; 1.27] |
| Fuller 2007a | 110 | 103502 | 440 | 442340 | 1.07 | [0.87; 1.32] |
| Fuller 2007b | 117 | 14192 | 547 | 65061 | 0.98 | [0.80; 1.20] |
| Kristenson 2013 | 119 | 156381 | 186 | 161655 | 0.66 | [0.53; 0.83] |
| Lanzetti 2017 | 0 | 1270 | 1 | 1310 | 0.34 | [0.01; 8.44] |
| Soligard 2010 | 6 | 6022 | 123 | 56575 | 0.46 | [0.20; 1.04] |
| Random effects model | | | | | **0.88** | **[0.73; 1.05]** |
| Heterogeneity: $I^2 = 63\%$, $\tau^2 = 0.0416$ | | | | | | |
| **Strain** | | | | | | |
| Almutawa 2014 | 45 | 1901 | 49 | 1481 | 0.72 | [0.48; 1.07] |
| Aoki 2010 | 36 | 37842 | 55 | 67514 | 1.17 | [0.77; 1.78] |
| Bjørneboe 2010 | 88 | 74612 | 258 | 186929 | 0.85 | [0.67; 1.09] |
| Ekstrand 2006 | 59 | 58512 | 61 | 24362 | 0.40 | [0.28; 0.58] |
| Ekstrand 2011a | 492 | 269550 | 2416 | 906386 | 0.68 | [0.62; 0.75] |
| Fuller 2007a | 120 | 103502 | 525 | 442340 | 0.98 | [0.80; 1.19] |
| Fuller 2007b | 66 | 14192 | 298 | 65061 | 1.02 | [0.78; 1.33] |
| Kristenson 2013 | 124 | 156381 | 251 | 161655 | 0.51 | [0.41; 0.63] |
| Lanzetti 2017 | 13 | 1270 | 14 | 1310 | 0.96 | [0.45; 2.04] |
| Soligard 2010 | 13 | 6022 | 168 | 56575 | 0.73 | [0.41; 1.28] |
| Random effects model | | | | | **0.76** | **[0.61; 0.93]** |
| Heterogeneity: $I^2 = 80\%$, $\tau^2 = 0.0824$ | | | | | | |
| **Other** | | | | | | |
| Almutawa 2014 | 15 | 1901 | 14 | 1481 | 0.83 | [0.40; 1.73] |
| Aoki 2010 | 21 | 37842 | 38 | 67514 | 0.99 | [0.58; 1.68] |
| Bjørneboe 2010 | 26 | 74612 | 110 | 186929 | 0.59 | [0.39; 0.91] |
| Ekstrand 2012 | 10 | 274305 | 41 | 905219 | 0.80 | [0.40; 1.61] |
| Fuller 2007a | 68 | 103502 | 234 | 442340 | 1.24 | [0.95; 1.63] |
| Fuller 2007b | 64 | 14192 | 268 | 65061 | 1.09 | [0.83; 1.44] |
| Kristenson 2013 | 33 | 156381 | 55 | 161655 | 0.62 | [0.40; 0.95] |
| Lanzetti 2017 | 4 | 1270 | 4 | 1310 | 1.03 | [0.26; 4.12] |
| Soligard 2010 | 8 | 6022 | 89 | 56575 | 0.84 | [0.41; 1.74] |
| Random effects model | | | | | **0.89** | **[0.71; 1.11]** |
| Heterogeneity: $I^2 = 42\%$, $\tau^2 = 0.0492$ | | | | | | |

Heterogeneity: $I^2 = 70\%$, $\tau^2 = 0.0515$

Test for subgroup differences: $\chi^2_3 = 3.82$, df = 3 ($p = 0.28$)

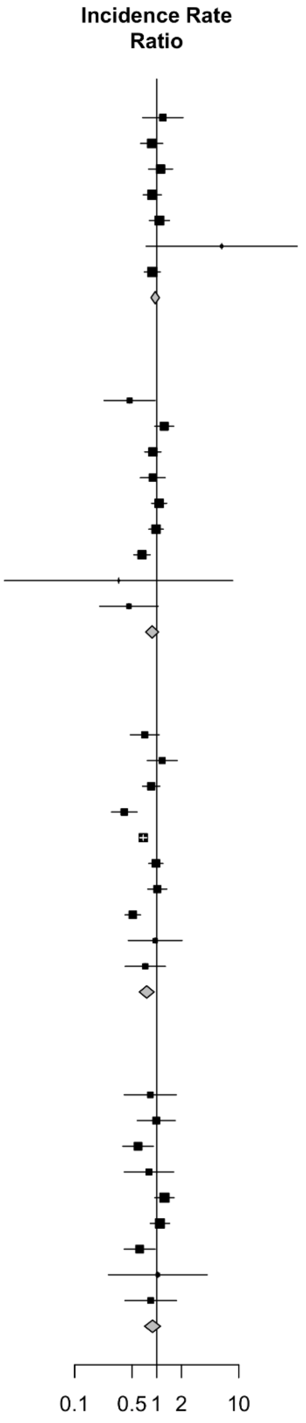

Supplementary figure 21: Sensitivity analysis with only studies with lowest risk of bias (9 out of 9 stars in Newcastle-Ottawa Scale) included. Forest plot of the incidence of overall injuries on artificial turf compared to grass stratified by sex.

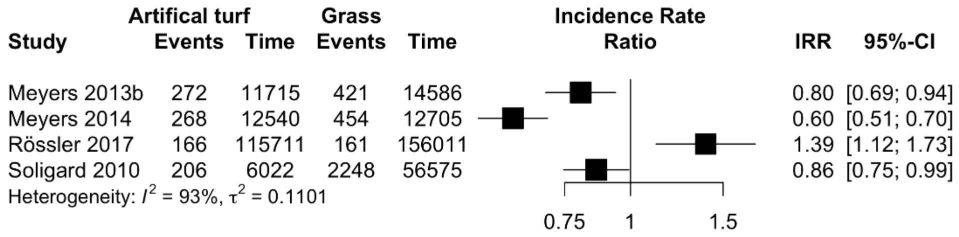

Supplementary figure 22: A) Funnel plot assessing the publication bias for studies comparing artificial turf to grass. Eggers test intercept 0.6 (95% CI: -3.3 to 4.6) B) Funnel plot assessing the publication bias for studies comparing artificial turf to other playing surfaces. Eggers test intercept -4.0 (95% CI: -25.1 to 17.2). We did not find evidence supporting the presence of a publication bias in the included studies.

A)

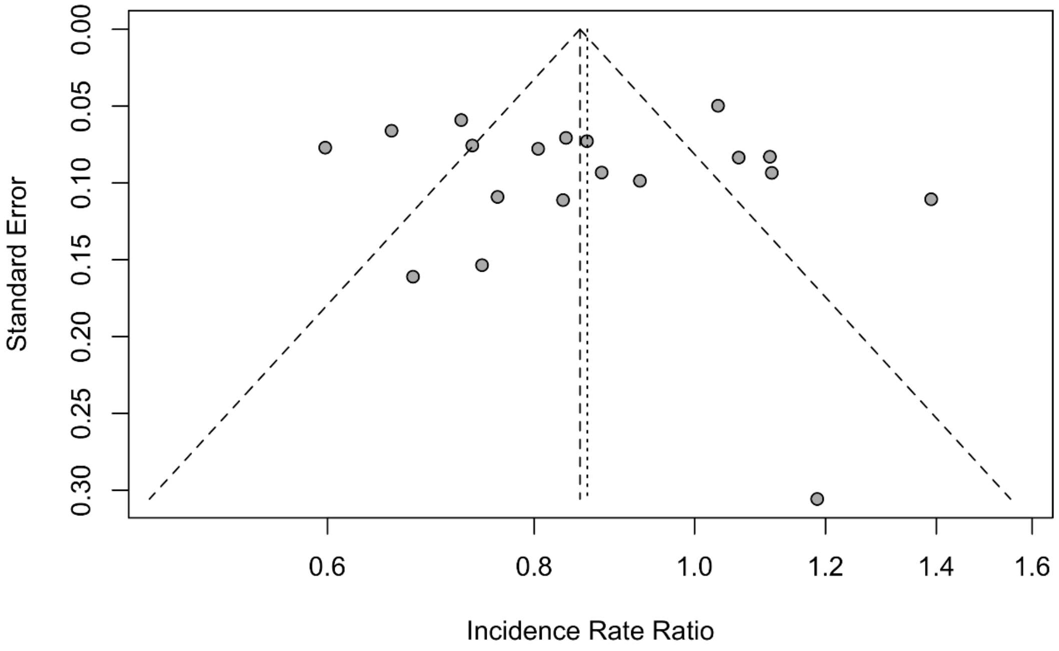


B)

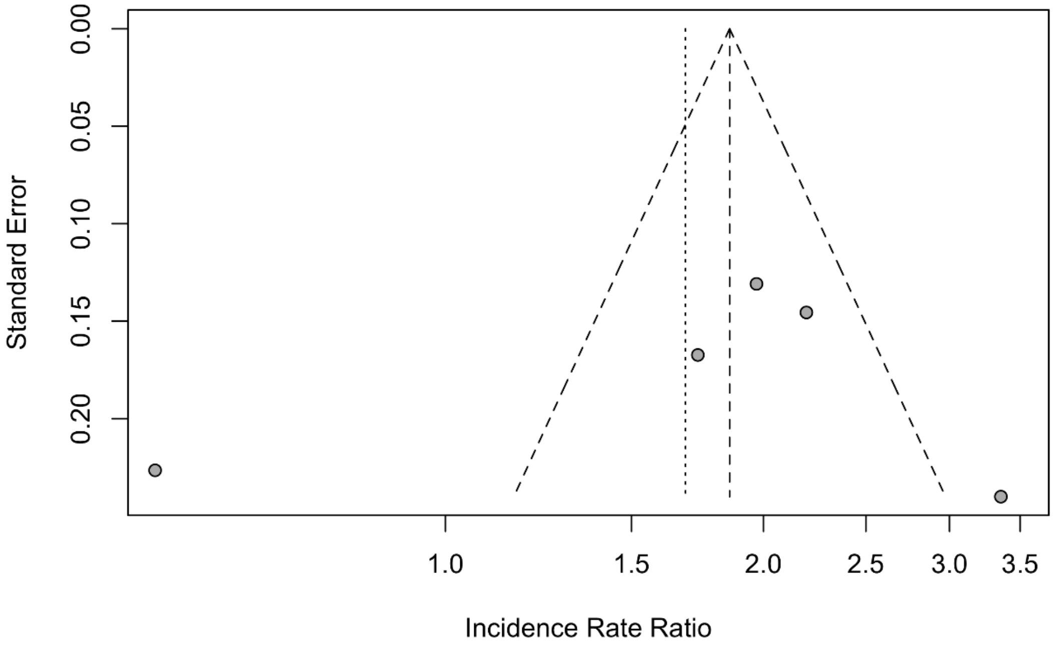

Supplement: Supplementary Figure S1–S22 [file mmc3.pdf]
